# Peptide-Assisted Nucleic Acid Delivery Systems on the Rise

**DOI:** 10.3390/ijms22169092

**Published:** 2021-08-23

**Authors:** Shabnam Tarvirdipour, Michal Skowicki, Cora-Ann Schoenenberger, Cornelia G. Palivan

**Affiliations:** 1 Department of Chemistry, University of Basel, Mattenstrasse 24a, 4058 Basel, Switzerland; shabnam.tarvirdipour@unibas.ch (S.T.); michaljerzy.skowicki@unibas.ch (M.S.); 2 Department of Biosystem Science and Engineering, ETH Zurich, Mattenstrasse 26, 4058 Basel, Switzerland; 3 NCCR-Molecular Systems Engineering, BPR1095, Mattenstrasse 24a, 4058 Basel, Switzerland

**Keywords:** amphiphilic peptides, non-viral gene delivery, nanocarrier, peptide self-assemblies, stimuli responsive

## Abstract

Concerns associated with nanocarriers’ therapeutic efficacy and side effects have led to the development of strategies to advance them into targeted and responsive delivery systems. Owing to their bioactivity and biocompatibility, peptides play a key role in these strategies and, thus, have been extensively studied in nanomedicine. Peptide-based nanocarriers, in particular, have burgeoned with advances in purely peptidic structures and in combinations of peptides, both native and modified, with polymers, lipids, and inorganic nanoparticles. In this review, we summarize advances on peptides promoting gene delivery systems. The efficacy of nucleic acid therapies largely depends on cell internalization and the delivery to subcellular organelles. Hence, the review focuses on nanocarriers where peptides are pivotal in ferrying nucleic acids to their site of action, with a special emphasis on peptides that assist anionic, water-soluble nucleic acids in crossing the membrane barriers they encounter on their way to efficient function. In a second part, we address how peptides advance nanoassembly delivery tools, such that they navigate delivery barriers and release their nucleic acid cargo at specific sites in a controlled fashion.

## 1. Introduction

Introducing exogenous nucleic acids into human target cells has been receiving a great deal of attention for the treatment of several human diseases, in particular cancer and other genetic disorders. Quite recently, a new treatment involving gene editing CRISPER has made a mark by using mRNA encoding Cas [1,2]. In face of the worldwide coronavirus pandemic, mRNA has moved into the limelight as vaccine and many companies are working on other mRNA vaccines and therapeutics [3,4]. Both vaccines and disease intervention involve delivering nucleic acids to intracellular locations on a path strewn with obstacles. To ultimately accomplish modification of protein expression by replacing or adding missing or defective genes, regulating gene expression at the RNA level (e.g., gene silencing by RNA interference, modification of RNA processing), controlling microRNA activity or by genome editing and reprogramming of cells, nucleic acids face a number of challenging barriers. Hence, despite a broad range of possible therapeutic approaches, the clinical success of gene therapy has yet to meet the expectations. The lack of efficacy and issues with clinical safety, in particular with viral vectors, which make up about 70% of vectors used in gene therapy, are the main reasons gene delivery systems fail in clinical trials [5,6]. This has led to the emergence of non-viral vector systems, such as liposomes and polymer supramolecular assemblies with better biological safety. However, their efficacy is predominantly hampered by insufficient localization of the therapeutic agents at the site of interest, both at the extracellular and intracellular level [7]. Owing to their remarkable potency, selectivity and low toxicity, peptides offer ideal alternatives to overcome these hurdles [8]. In addition, advancements in nanosystems continue to open new avenues for an efficient delivery of therapeutics and, thus, nanotechnology has become a favored tool in medicine [7].

Nanocarriers based on their size, shape, charge, and surface chemistry are internalized by target cells through different pathways including clathrin-mediated endocytosis, caveolae- or cholesterol-mediated endocytosis, phagocytosis, and macropinocytosis [9,10]. After entering cells by endocytosis, nanocarriers usually remain sequestered in corresponding transport vesicles and their fate depends on the endocytic pathway but also on the physicochemical properties of the nanocarriers. Endosomal sequestration consists of multiple membrane fusions, in which the endocytic vesicles sequentially merge with early and late endosomes, proceeding all the way to the lysosomal compartment [10]. A constant decrease in intravesicular pH and increase in digestive enzymatic content throughout this pathway have a major impact on the stability of payloads and, subsequently, on efficacy [10,11]. These limitations have led to the search for strategies that can properly protect the macromolecular drugs from degradation and specifically target the major subcellular compartments. Furthermore, a boost of discovery research for the better understanding of intracellular trafficking routes highlight the need for carriers that overcome the barriers associated with the delivery to the intracellular site of action [11].

The major shortcomings of most commonly used non-viral nucleic acid delivery systems, such as lipoplexes and polyplexes include nonspecific distribution, inefficient cytoplasmic delivery, and organelle targeting. In contrast, peptide-based nanocarriers, e.g., peptide nanoparticles, also called peptiplexes, or peptidic multicompartment micelles, and nano-assemblies equipped with peptides hold great promise as delivery platforms, since they can be tweaked to facilitate penetration of cell membranes and to localize to distinct subcellular compartments. In addition, peptides are easy to synthesize with a desired bioactivity, and, by multivalent presence, endow the nanocarrier with high avidity for the target [10,12]. Owing to the highly specific targeting capacity of corresponding peptides, therapeutic nanocarriers are able to pass through the cell membrane and reach the specific tissue and cells which results in enhanced intracellular distribution and extended therapeutic window [13]. Furthermore, smart delivery systems are promising options to provide solutions related to uncontrolled release of payloads: besides a biocompatible nanocarrier and suitable targeting moieties, these platforms include stimulus-responsive elements which endow them with triggered cargo release [14].

The concept of using peptides as targeting moieties for therapeutic and diagnostic purposes has created new avenues for modern pharmaceutical industries [13,15]. Although clinical progress in the application of peptides, alone or combined with nano-assemblies, is slowly moving forward, large investments and wide-ranging research efforts confirm their promising potential as a delivery platform for therapeutic systems. Increased interest in smart nanocarrier design with particular focus on, but not limited to, cancer therapy with the aim of precision medicine application has boosted this unique class of pharmaceutical compounds into high demand [16,17,18].

In this review, we discuss various types of membrane active and stimuli responsive peptides with regard to their role in refining different nanocarriers for gene delivery applications. As peptides take center stage, we do not cover predominantly lipidic nor inorganic nanoparticle gene delivery systems. We describe properties of peptides that promote site-specific localization of nucleic acids and of peptide-based nano-assemblies. Then, we lay the emphasis on peptide designs that confer stimuli-responsiveness upon nanosystems with the aim to control payload release. Targeting, controlling, and stimuli-responsive peptides advance nanosystems from non-specific carriers of nucleic acids to smart site-specific gene delivery systems.

## 2. Peptide-Guided Delivery of Nucleic Acids across Biological Barriers

**Figure 1:**
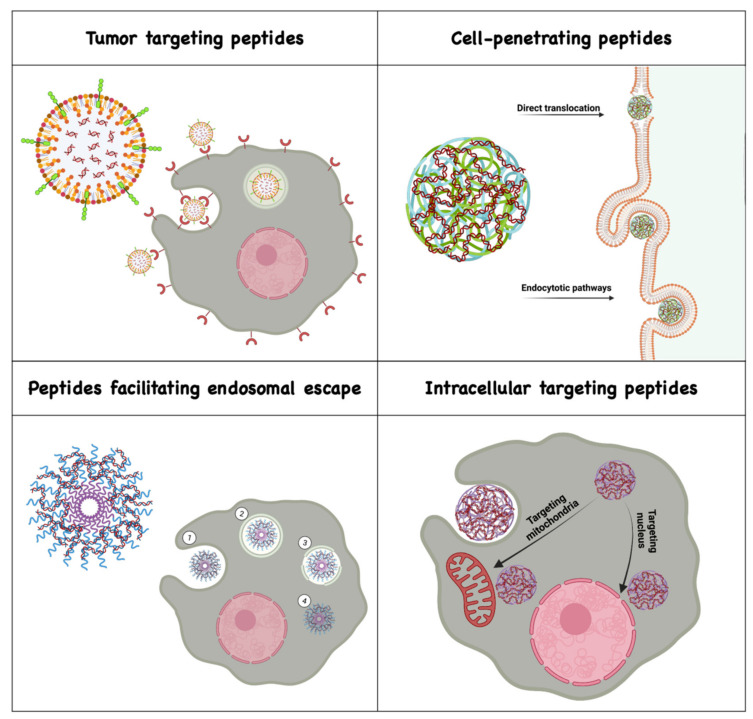
Classes of membrane active peptides facilitating the delivery of nucleic acid across biological barriers. Created with BioRender.com (Access to BioRender: June–July).

Membrane active peptides interact with cellular membranes by traversing them, disrupting them or by residing at the membrane interface and fusing with them [19]. They are known to overcome site-specific delivery barriers and facilitate intracellular delivery of various bioactive cargos with low cytotoxicity [19,20]. Although there is a wide variety of membrane-active peptides, here we mainly discuss peptides for targeting nucleic acid delivery systems to specific cells and tissues, and peptides that assist in the delivery of nanocarriers across membrane barriers, such as cell penetrating peptides (CPPs), peptides facilitating endosomal escape and those that target nanocarriers to subcellular organelles (Figure 1).

### 2.1. Tumor-Targeting Peptides

The ability of peptides to mediate translocation across membranes, traffic to desired sites, as well as executing many fundamental cellular functions made them promising candidates for targeting [21]. Owing to the high mortality related to cancer, substantial research investments have been made over the past decades in order to develop specific cancer diagnostics and treatments that improve survival rate [22]. The aberrant proliferation of tumor cells, accompanied by the up-regulation of their molecular markers result in high levels of specific receptors in the tumor and its microenvironment [23]. Thus, tumor-targeted delivery methods incorporate peptides or antibodies that are selective to the receptors overexpressed on the tumors [24]. Selective targeting of these tumor-associated markers promises the accurate targeting of signaling pathways that are dysregulated in the tumor [25].

**Figure 2:**
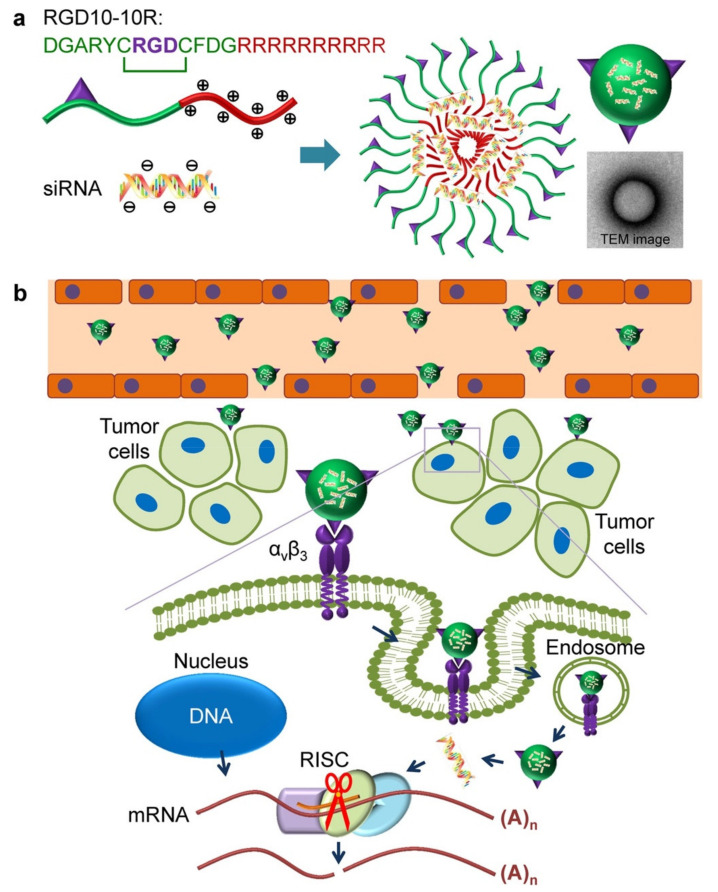
Schematic representation of (**a**) fabrication of the RGD10-10R/siRNA complex, (**b**) tumor-targeted siRNA delivery involving ligand/receptor interactions. siRNAs accumulated in the tumor tissue and then entered the tumor cells in a receptor (αvβ3)-mediated endocytosis (RME) manner in vitro. After being internalized by cells, peptide/siRNA complexes escaped from the endosomes/lysosomes. Then, siRNAs were released from the complexes and loaded by RNA-induced silencing complex (RISC). Targeted messenger RNA complementary to the guide strand (antisense strand) of siRNA was selected and cleaved by argonaute protein. Reprinted with permission from [31]. Copyright 2015 Springer Nature.

Although the use of antibodies to target tumors has become highly successful both in tumor diagnosis and therapy, some deficiencies associated with antibodies, such as inadequate pharmacokinetics and limited tissue accessibility, as well as impaired interactions with the immune system limit their clinical application. Compared to antibodies and other tumor-targeting ligands, peptides offer better cell or tissue penetration, high affinity and targeting specificity, low immunogenicity, high stability, and improved pharmacokinetics by chemical modifications [26]. Tumor-targeting peptides, usually comprising less than 50 amino acids, are synthesized naturally or artificially [27,28]. For example, peptide sequences containing an arginine-glycine-aspartic acid (RGD) motif are among the most prominent targeting moieties for non-viral delivery systems [29]. The strong affinity of the RGD motif for integrin receptors expressed on vascular endothelial cells and overexpressed on many cancer cells [30] facilitates cell attachment and uptake of nanocarriers by receptor-mediated endocytosis (Figure 2) [31].

Likewise, the synthetic nonapeptide LyP-1 is an example of a tumor targeting peptide that can selectively bind to its primary receptor p32 protein overexpressed in various tumor-associated cells and atherosclerotic plaque macrophages [32]. Binding leads to proteolytic cleavage of LyP-1 into a truncated version whose exposed C-terminal CendR motif becomes active and triggers binding to NRP1 and/or NRP2 cell surface receptors [32,33]. This interaction promotes cellular internalization of LyP-1 and its bioconjugates. NRP1/2 also mediates transfer to the nucleus, which makes LyP 1-based delivery systems more effective in imaging and treatment of diseases [34]. An overview of different tumor-targeting peptides developed for cancer gene therapy is presented in Table 1.

### 2.2. Cell-Penetrating Peptides

**Figure 3:**
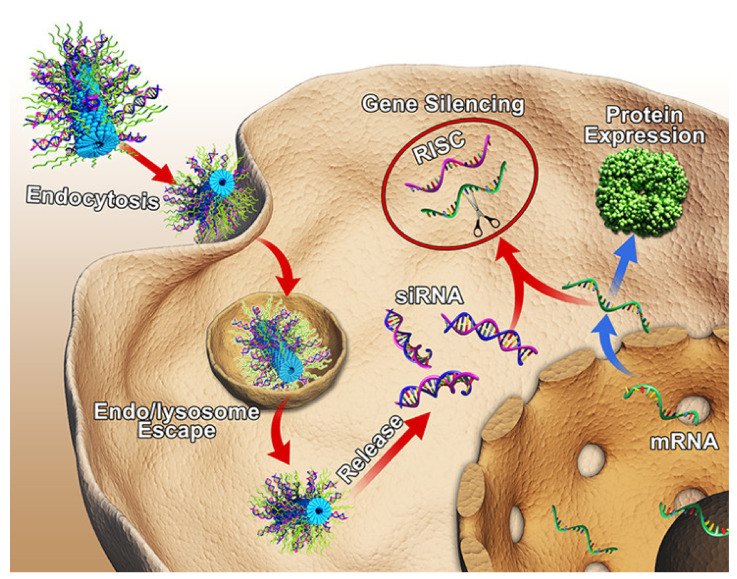
Schematic illustration of cell penetrating TAT peptides complexed with siRNA and integrated into modified tobacco mosaic virus (TMV) for virus-inspired gene silencing. Reprinted with permission from [52]. Copyright 2018 American Chemical Society.

Cell-penetrating peptides (CPPs) are short peptides (less than 30 amino acids) derived from naturally occurring proteins, designed de novo or a combination of both [48]. CPPs by virtue of their ability to permeate the cell membrane in an innocuous manner provided a means for successful cellular entry and intracellular trafficking of a wide variety of cargos including nucleic acids (Figure 3) [49,50,51,52,53]. In addition to sequence length, charge and amphipathicity are the main structural parameters determining internalization but also cargo interactions. Penetration of nucleic acids across the cell membranes is a key step in gene delivery and paves the way for an efficient gene therapy [52]. Nucleic acids can be conjugated to CPPs, either by non-covalent complex formation or by covalent bonds [54]. CPPs promote the intracellular distribution of these membrane-impermeable therapeutic molecules without destroying the integrity of cellular membranes and, thus, widen the therapeutic window of cargos [13].

CPPs can be classified according to their physicochemical properties as cationic, amphipathic, and hydrophobic, which largely impacts the type of cell-membrane interactions and uptake mechanism [55]. Extensive literature is available on the structure–activity relationship of CPPs [56,57,58,59]. Examples of CPPs classified according to their physicochemical properties and the genetic cargo they delivered are summarized in Table 2.

Cationic CPPs show a high affinity for negatively charged cell membranes because of electrostatic interactions and, thus, internalize into the cell through a receptor-independent mechanism. The key factors determining the activity of cationic CPPs are the number and position of positively charged amino acids in their structure [57]. TAT and penetratin, the first cationic CPPs discovered, have been widely used to promote cellular uptake and transfection efficiency of various lipid-, polymer-, and peptide-based nanocarriers [119,120,121]. Accordingly, several artificial homopolymers of arginine and lysine peptides have been developed to effectively translocate cargo across the membrane [122,123]. Notably, the rate of cell uptake and subsequently transfection efficiency was higher for arginine-rich peptides compared to polylysines [123,124,125].

Although most naturally occurring CPPs are cationic, the major class of CPPs is amphipathic [48]. Amphipathic CPPs consist of polar and non-polar (rich in hydrophobic) amino acid regions that are able to fold into α-helical and β-sheet-like structures. The secondary structure might change in response to different physiological conditions which, in turn, affects their penetration ability [57]. Prominent representatives of amphipathic CPPs are various variants of N-Methylpurine DNA Glycosylase or MPG, where amphiphilicity is a leading factor for their translocation across the membrane [126]. MPGs undergo a conformational transition from unordered into a folded state upon their interaction with membrane phospholipids mediated by polar residues. The resulting β-sheet conformation governed by the hydrophobic domain of MPG lead to transient pore-formation in the cell membrane, which in turn enable the MPG/cargo complexes direct penetration across the membrane independent of endocytosis [126,127]. In addition to MPGs’ function in promoting cellular internalization, it is well known for its strong electrostatic interactions with oligonucleotides [128]. Consequently, MPG family members form stable noncovalent nanocomplexes with nucleic acids that enter cells independently of the endosomal pathway. Accordingly, MPG has shown to efficiently deliver small interfering RNA (siRNA) and plasmid DNA (pDNA) into cultured cell lines [129]. Transportan and its analogs NickFect and PepFect are other examples of amphipathic peptides that can condense pDNA and siRNA into stable nanocomplexes [130,131,132]. Although their hydrophobicity appears to be responsible for the nanocomplexes’ stability, the pH-induced change of their charge plays a key role in promoting oligonucleotide condensation and high delivery efficiency.

Hydrophobic CPPs with low positive or negative net charge are less common and their uptake mechanism is not well understood. For example, natural C105Y, K-FGF, and Bip peptide belong to this group and their non-polar amino acids’ affinity to the hydrophobic domain of cell membranes mediate their translocation [48].

### 2.3. Peptides Facilitating Endosomal Escape

Endosomal escape is a crucial step in improving intracellular delivery and efficiency of nucleic acids [133]. Following endocytosis as the major uptake route for many peptide-based nanocarriers, most internalized nanocarriers significantly suffer from their interaction with endosomal membrane which leads to their entrapment and eventually their enzymatic degradation in the lysosomal compartment [134,135]. Peptides are the most promising candidates for promoting endosomal escape [136,137]. In particular, pH-sensitive peptides that at physiological pH adopt a random coil structure, transform to an α-helical conformation able to induce membrane pore formation in the acidic environment of endosomes [138]. Similarly, studies on viruses escaping the lysosome have shown that some viral peptides change from a hydrophilic ring structure to a hydrophobic spiral structure which will target the core of the bilayer and destroy the stability of the membrane [139]. Peptide development aims at facilitating escape from the endosome via pore formation, the “proton sponge effect”, or conformational changes.

#### 2.3.1. Fusogenic Peptides

**Figure 4:**
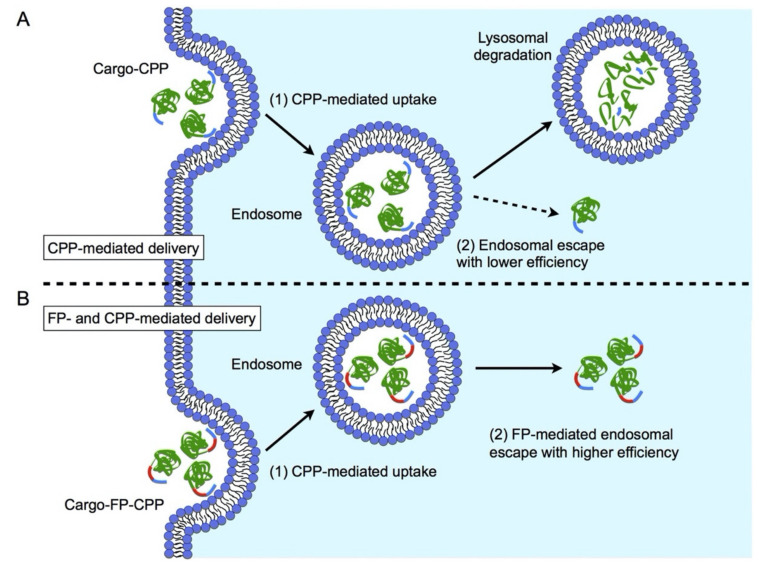
Schematic representation of FP and CPP-mediated delivery. (**A**) A conventional CPP-mediated delivery. (1) (**A**) cationic CPP (blue) interacts electrostatically with the anionic cell-surface, and a CPP-fused cargo (green) is internalized into the endosome by endocytosis. (2) Because the endosomal escape efficiency of CPP is low, the cargo-CPP is subjected to lysosomal degradation. (**B**) FP- and CPP-mediated delivery. (1) An FP (red)- and CPP-fused cargo is internalized into the endosome by endocytosis. (2) Because the efficiency of FP-mediated endosomal escape is relatively high, the cargo-FP-CPP is efficiently transferred from the endosome to the cytoplasm. Reprinted with permission from [142]. Copyright 2017 Elsevier.

Fusogenic peptides (FPs) are short peptides with the potential to promote membrane destabilization and delivery of nucleic acids to the cytosol and/or the nucleus [140]. Fusogenic peptides consist of hydrophilic and hydrophobic domains that are able to form helical structures at endosomal pH. This allows for direct engagement with the endosomal membrane upon which further energetically favorable conformational changes induce pore formation in the membrane. Disruption of the bilayer eventually leads to endosomal escape of nanocarriers equipped with FPs and release of cargos to the cytoplasm (Figure 4). Overcoming the endosomal membrane barrier presents an important role in facilitating nucleic acids localization to distinct subcellular compartments as their site of action [54]. However, before integrating a fusogenic peptide into gene delivery systems, the cellular uptake mechanism should be considered. Since the fusogenic activity of these peptides is due to a pH-dependent shift in conformation, non-acidic endocytotic pathways, such as caveolae-mediated endocytosis and macropinocytosis will revoke their membrane lytic activity [141].

Fusogenic peptides are either derived from the transduction domain of proteins that interact with cell membranes such as HA2, INF7, and melittin or are synthetic amphipathic peptides that can penetrate membranes [141,143].

Wild-type HA2(1–23) peptide and a glutamic acid-enriched analogue (INF7) from influenza virus hemagglutinin are the oldest and best studied fusogenic peptides used for gene delivery [144,145,146,147,148,149]. These peptides, based on the protonation of their acidic residues upon a decrease in pH, assume a helix structure and consequently promote the endosomal escape, which, in turn, results in enhanced transfection efficiency [150,151].

Melittin, a cationic amphipathic peptide composed of 26 amino acids, is derived from the venom of the honey bee Apis mellifera. Melittin with its predominantly hydrophobic 20 N-terminal amino acids and hydrophilic C-terminus acts mainly like a natural detergent on the membrane and is well known for its cytolytic activity [152]. Oligomerization of this peptide results in the formation of transmembrane channels which lead to osmotic cell lysis [153]. Owing to its cytotoxicity, the use of melittin as an agent to promote gene delivery in transfected mammalian cells is limited. More recently, less toxic melittin analogues that retained their ability to escape from the endosome were shown to enhance the efficiency of non-viral gene delivery systems [154,155,156].

A number of pH-responsive synthetic amphipathic peptides mimic the fusogenic activity of virus-derived peptides. The most prominent representatives consist of non-polar alanine-leucine-alanine repeating units with considerable repetitive content of either glutamic acid, lysine, or arginine, and are named GALA, KALA, or RALA, respectively [157,158,159,160,161]. Likewise, upon protonation at endosomal pH (5.0), they assume an amphipathic α-helical conformation which is associated with a significant affinity for binding to phospholipid membranes. As a consequence, pore formation, membrane fusion, and/or lysis are induced. Their membrane lytic activity explains extensive utilization of these fusogenic peptide in modulating non-viral gene delivery systems [76,162,163,164,165,166]. Similar to GALA, JTS-1, a negatively charged amphipathic peptide with strong nonpolar amino acids in the hydrophobic domain and glutamic acid residues in the hydrophilic domain is able to form an α-helical structure [167]. Owing to the endosomolytic capacity of JTS-1-modified carriers, improved transfection activity was reported in several studies [168,169].

Taking advantage of the fusogenic properties of peptides, alone or in combination with other advantageous attributes, promotes the efficacy of peptide-based nanocarriers for traversing membranes and, thereby, improves their therapeutic effects. Yet, there is a strong need for systematic study of different fusogenic peptides under similar conditions in order to elucidate the mechanisms and pin down the parameters that ultimately will maximize therapeutic outcome. This comprehensive comparison of fusogenic peptides will allow for designing a robust, widely applicable delivery system.

#### 2.3.2. Histidine-Rich Peptides

**Figure 5:**
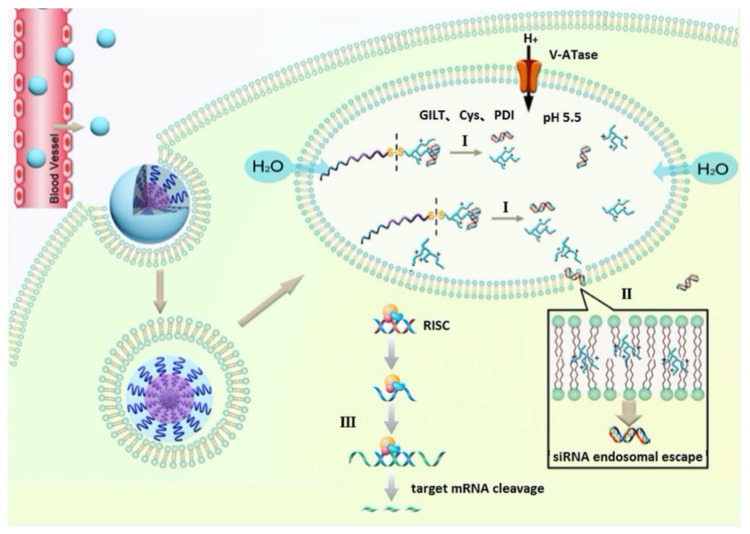
Schematic illustration of the intracellular trafficking of mPEG-b-PLA-Phis-ssPEI and siRNA complexes. After internalized by tumor cell, (**I**) the complexes rapidly disassemble to release siRNA and free polyethylenimine (PEI) molecules in response to the acidic and reductive microenvironment, (**II**) efficiently escape from the endosome, facilitated simultaneously by cleaved PEI chains inducing membrane destabilization, the “proton sponge effect” of polyhistidine and polyethylenimine, as well as the relative small size of after disassembly, (**III**) achieve efficient gene silencing by cytosolic target mRNA cleavage. Adapted with permission from [179]. Copyright 2018 Elsevier.

The combination of being able to condense nucleic acids and at the same time promote endosomal escape spurred efforts to incorporate histidine-rich amphipathic peptides into various gene delivery systems [170]. As described for fusogenic peptides, histidine residues that become protonated during acidification of the endosome interact with negatively charged membrane lipids (Figure 5) and destabilize the membrane [171]. Histidylation of different non-viral vectors among which polylysine was the first example, was found to increase the buffering capacity of vectors [172]. Substituting several lysines with histidines turns polylysine into a successful gene delivery vector with enhanced transfection efficiency [173,174,175,176,177,178].

Influenza-derived, histidine-rich H5WYG peptide that is capable of traversing intracellular barriers to deliver nucleic acids, is another well-known example that raised great interest. This pH sensitive peptide has been extensively applied to improve the gene delivery efficacy of different polymeric, peptidic, and lipid-based carriers [180,181,182,183,184,185,186].

Cationic LAH4 is another widely studied histidine-rich peptide where the protonation of the imidazole groups invokes chloride ion, as well as proton influx into the endosome, creating a hypertonic environment. Although the so-called “proton sponge effect”, i.e., the osmotic influx of water triggering endosome lysis, has been recognized as the primary route of endosomal escape, other mechanisms also exist [187,188]. Changes in pH modulate the amphipathicity and membrane topology of LAH4 and derivatives: they are transmembrane at neutral pH, whereas under acidic conditions, LAH4 peptides align parallel to the phospholipid bilayer surface [189,190]. The interactions of the peptides with the bilayer interface eventually result in pore-formation [191] and membrane lysis [192], thereby modulating nucleic acid delivery. Inspired by the ability to enhance the efficiency of several gene delivery systems, researchers in the past extensively used LAH4 peptides as targeting moiety [187,193,194,195,196,197,198]. By now, there is a growing number of peptidic, as well as polymeric and lipidic carriers that utilize other histidine-rich moieties, such as O_10_H_6_ [199], MS(O_10_H_6_) [200,201], histidine-rich Tat peptide [202,203,204], or His6 RPCs [205,206] to develop new promising gene delivery strategies. The possibility of intracellular delivery of nucleic acids in a nontoxic manner, which is a necessary prerequisite for gene therapy, has opened interesting perspectives in non-viral gene delivery. Nevertheless, there are many unanswered questions regarding the precise capacity or trafficking routes involved in favoring endosomal escape [172]. Hence, a comprehensive screen and quantification of this step will provide further improvements for exploiting histidine-rich peptides in the field of gene therapy.

### 2.4. Peptides Assisting Delivery to Subcellular Organelles

A major focus in gene therapy is delivering nucleic acids to those intracellular compartments where they are therapeutically most effective. By escaping the endosome, siRNA and mRNA cargos arrive at their final destination, the cytosol, whereas DNA cargoes require translocation to the nucleus or to mitochondria. Intracellular targeting peptides serve a promising approach to specifically direct their cargo to the respective organelles and ensure membrane interactions that support delivery. Obviously, such peptides are particularly favorable candidates to be integrated into gene delivery systems [10,207]. The degree of translocation enhancement depends on the characteristics of both, delivery system and targeting peptide [208]. In the following sections, we address the mechanisms of intracellular nanoparticle trafficking and provide examples employing intracellular targeting peptides to ensure nuclear and mitochondrial targeting of nucleic acids.

#### 2.4.1. Nuclear Localization Signals

**Figure 6:**
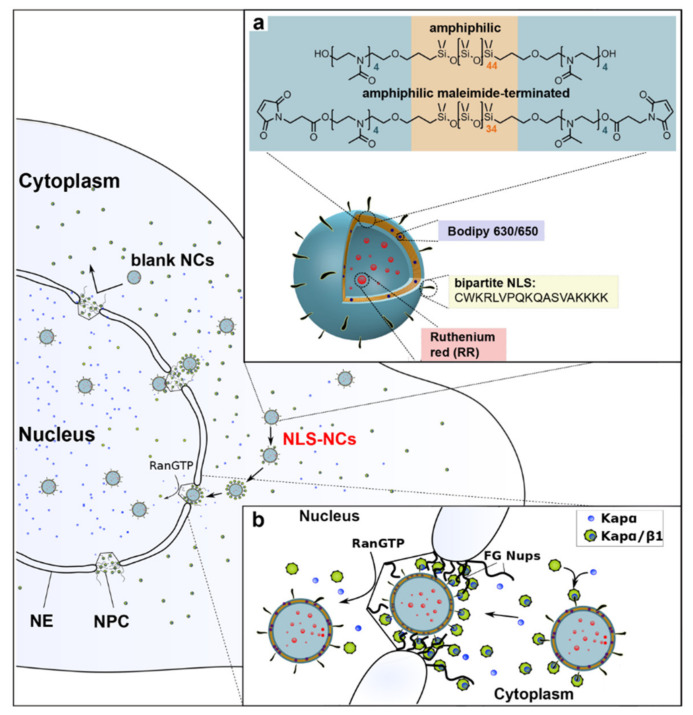
Organelle-specific targeting of polymersome NCs into the cell nucleus. (**a**) NLS-NCs self-assemble from amphiphilic PMOXA-PDMS-PMOXA triblock copolymers. Two model compounds are used to test for nuclear delivery: Ruthenium red (RR) that is encapsulated within the NLS-NC lumen, and Bodipy 630/650 that incorporates into its polymeric membrane. (**b**) The nuclear transport mechanism involves Kapα•Kapβ1 that (1) authenticates NLS-NCs for selective NPC transport, (2) binds to FG Nups, and (3) releases NLS-NCs into the nucleus upon binding RanGTP. Reprinted with permission from [213]. Copyright 2020 National Academy of Sciences.

Nucleocytoplasmic transport is major consideration for effective non-viral gene delivery [207,209]. Once inside the cell, most DNA must translocate into the nucleus where they can be either transcribed into the messenger RNA (mRNA) or interfere with transcription and RNA processing [210,211]. Nuclear localization signals (NLSs) are short peptide motifs rich in arginine, lysine, or proline that mediate nuclear translocation and when attached to foreign macromolecules or nanocarriers, deliver them to the nucleus [212]. For example, polymersomes, artificial vesicles resulting from self-assembly of amphiphilic copolymers, bypass the nuclear pore complexes (NPCs) that regulate transport into and out of the nucleus and deliver payloads directly into cell nuclei (Figure 6) [213]. Active transport of macromolecules to the nucleus is carried out by interactions of the NLS with importin receptors (karyopherins) and specific proteins of the NPC [214,215].

In view of the fact that the nuclear membrane is the main barrier restricting transgene expression of most non-viral carriers, gene therapy is the obvious field for the application of nuclear targeting peptides [216]. The significance of incorporating NLS peptides into non-viral delivery systems that can adequately favor the genetic materials release into the nucleus manifests itself by expanding case studies [207,217]. Hereby, positively charged NLS peptides either are attached to the negatively charged DNA via electrostatic interactions or are covalently coupled to the phosphate backbone of the DNA or to the condensing agent of the non-viral vector [216].

A frequently used NLS is derived from the large tumor antigen of Simian virus 40, SV40 (PKKKRKV). The positive charges of SV40 NLS peptide not only help in DNA condensation, but also mediate nuclear targeting [218]. Consistently, addition of the SV40 NLS peptide and its derivatives enhanced the transfection efficiency of many non-viral carriers [219,220,221,222].

Another interesting NLS is M9, a 38 amino acid peptide derived from heterogeneous ribonucleoprotein A1 (hnRNP A1) which is a major nuclear pre-mRNA binding protein. M9 is responsible for ferrying hnRNP A1 into the nucleus and also contains a nuclear export sequence (NES) [223]. Owing to its rather low positive charge, M9 is relatively poor at condensing DNA. On the other hand, it strongly interacts with its known receptor transportin 1 [224]. Therefore, utilizing the nuclear import effect of M9 in combination with positively charged biomaterials that condense the DNA offers great potential for gene therapy [142,225,226]. Furthermore, an NLS sequence derived from the HIV-1 viral protein (Vpr) promotes nuclear import through a karyopherin α–independent mechanism [227]. Examples for an enhanced transfection efficiency with Vpr-containing non-viral vectors are reviewed by Cartier and Reszka [217].

Other examples of targeting sequences that facilitate nuclear transport of exogenous DNA include Xenopus protein nucleoplasmin [228,229], adenoviral peptide (Ad) [217,230], human T-cell leukaemia virus (HTLV) [224], Epstein–Barr virus nuclear antigen (EBNA)-1 [231]. By overcoming intracellular barriers, these peptides greatly expand the perspectives in non-viral gene delivery.

#### 2.4.2. Mitochondrial Delivery

Mitochondria have their own genome whose mutations are associated with numerous disorders, such as cancer, diabetes, neurodegenerative diseases including Parkinson’s disease, and more recently infectious and autoimmune diseases [225,226]. Given the link between mitochondrial dysfunction and disease, targeting of therapeutic interventions to these subcellular organelles is of vital importance [232,233]. Efficient mitochondrial gene therapy requires nanocarriers that, once inside the cell, target mitochondria and ferry nucleic acids across the outer (OMM), as well as inner mitochondrial membrane (IMM) [234]. The hydrophobicity and negative charge of MMs require that for efficient mitochondrial delivery, negatively charged DNA be shielded by carrier molecules, such as peptides that have amphiphilic and cationic properties.

Many natural and artificial short peptides and polypeptides have mitochondrial targeting ability [235,236]. Typically, these peptides comprise hydrophobic (phenylalanine, tyrosine, isoleucine) and positively charged (D-arginine, lysine) amino acids. The development of mitochondrion-targeted delivery strategies involving mitochondrial targeting sequences (MTSs), which are typically tens of amino acids in length, mostly takes advantage of MM properties including the high negative potential (−160 to −180 mV) and the intrinsic protein import machinery. Although MTSs vary in length, they have in common an α-helical structure with an amphiphilic surface that mediates internalization by endogenous transmembrane transporters. However, because of their rather large size, low solubility and insufficient permeability across the plasma membrane, MTSs by themselves are not suitable for delivering exogenous nucleic acids.

In addition to MTSs that are recognized by translocators, several smaller peptides consisting of 4–16 cationic and hydrophobic residues efficiently target and permeate mitochondrial double membranes [235,237]. These mitochondria-penetrating peptides (MPPs), also known as mitochondrial CPPs (mtCPPs), typically appear to penetrate cellular membranes directly rather than by endocytosis [238]. Consequently, nanocarriers targeted by MPPs circumvent endosome/lysosome segregation, which also increases the chance of (gene) delivery to the mitochondria. In addition, MPPs appear to have marginal effects on mitochondrial membrane potential [239]

**Figure 7:**
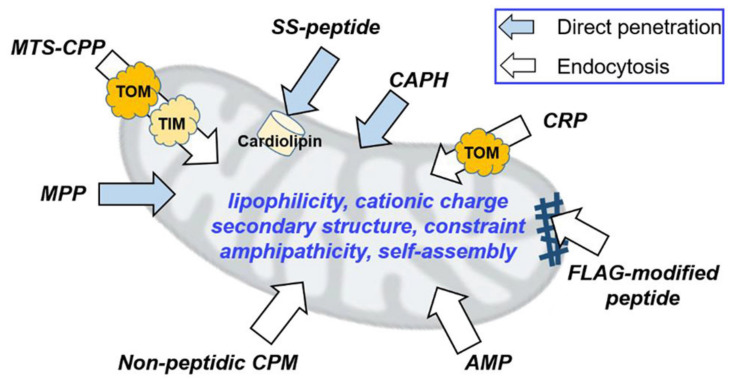
Mitochondrion-targeting peptides and peptidomimetics based on their structural classes and reported applications. Abbreviations: MTSCPP, MTS with cell-penetrating peptides; MPP, mitochondrion-penetrating peptides; SS-peptides, Szeto−Schiller peptides; CAPH, cationic amphiphilic polyproline helix; CRP, cysteine-rich peptides; FLAG-modified peptide, FLAG tag-based peptide that self-assembled into a nanofiber; AMP, peptides derived from antimicrobial peptides; CPM, nonpeptidic cell-penetrating motif; OMM, outer mitochondrial membrane; IMM, inner mitochondrial membrane; IMS, intermembrane space. Reprinted with modification from [233]. Copyright 2020 American Chemical Society.

Considering that cell and mitochondrial membrane barriers have distinct compositions and properties, a single peptide will not be able to mediate the crossing of both. Here, combining CPP activity and mitochondrial targeting can act synergistically to optimize delivery of peptide-based DNA nanoparticles to mitochondria (Figure 7) [233,240]. For example, a library of fusion peptides with mitochondria targeting (mtCPP1) and cell-penetrating properties (Pepfect14, a stearylated CPP forming ASO nanocomplexes with splice-correction activity in cells [241]) that self-assembled with antisense oligonucleotides (ASO) into complexes was successful in knocking down mitochondrial mRNA [242]. A combinatorial approach to develop mitochondrial gene expression was also pursued by incorporating an MTS into WRAP peptides (short tryptophan/arginine rich peptides; [243]) which formed nanocomplexes with plasmid DNA encoding the mitochondrial ND1 gene that were taken up by cells and targeted to mitochondria [244]. Systematic analysis of CPPs and MTSs revealed that while both types of peptides were rich in Ala and Arg, the latter included Leu, suggesting a role for Leu in targeting to mitochondria [245].

## 3. Peptide-Related Nano-Assemblies for Nucleic Acid Delivery

Peptides have great potential as self-assembly building blocks on account of primary and secondary structure variability. Depending on the design, they form various supramolecular assemblies, such as vesicles [246], micelles [247], nanotubes [248], nanofibers [249], or nanoribbons [250]. Choosing corresponding peptide building blocks allows for tuning size and shape of the nano-assembly to obtain improved nanocarrier properties including cargo loading and delivery. Moreover, the weak interactions involved in peptide self-assembly are sensitive to environmental conditions, enabling nano-assemblies to exhibit specific functionalities in response to different external stimuli, such as temperature, pH, redox state, enzymes, or even light. We first focus on examples of supramolecular assemblies where peptides represent the predominant building block of the nanocarriers or are integrated into the nanocarriers to improve their targeting and gene delivery properties, and then discuss examples with stimuli-responsiveness.

**Figure 8:**
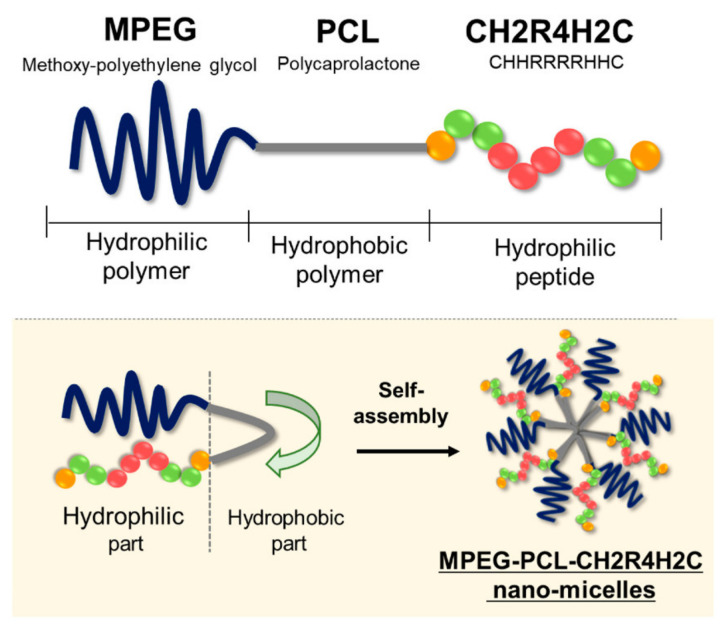
The structure of MPEG-PCL-CH2R4H2C and nano-micelle formation. Reprinted with permission from [252]. Copyright 2020 Multidisciplinary Digital Publishing Institute (MDPI).

Prominent structures that serve as nucleic acid carriers are micelles entrapping DNA during self-assembly, also called “micelleplexes” [251]. If purely peptidic, micelleplexes possess minimal cytotoxicity [20]. However, often peptides are used as a targeting or uptake-facilitating moieties associated with nanocarriers made out of different, less biocompatible materials. A micelle forming polymer-peptide conjugate used as an siRNA carrier has recently been reported as an effective tool in anti-metastasis cancer therapies (Figure 8) [252]. Methoxy-polyethylene glycol combined polycaprolactone conjugated with a cytoplasm-responsive peptide CH2R4H2C (MPEG-PCL-CH2R4H2C) was used to entrap anti-RelA siRNA (siRelA). RelA is a subunit of NF-κB involved in metastasis, especially cancer cell migration and invasion. The MPEG-PCL part of the conjugate was expected to improve blood retention and tumor accumulation and to facilitate micelle formation. Consistent with this notion, siRelA/MPEG-PCL-CH2R4H2C micelleplexes successfully delivered siRNA into cancer cells in a lung metastasis mouse model, causing inhibition of RelA accompanied by significant suppression of metastasis.

More recently, peptide-assisted polymeric micelles were used for inhibiting the mitotic cycle of prostate cancer cells by siRNA delivery in cell lines and in tumor-burdened nude mice [253]. The hydrophilic segments of acetal-polyethylene oxide-*b*-polycaprolactone (A-PEO-PCL) copolymers were chemically modified with a TAT peptide and a ligand for prostate-specific membrane antigen (DCL) to enhance targeting and cell penetration of self-assembled micelles loaded with siRNA and docetaxel (anti-cancer drug).

Other small molecules, for example a palmitoyl chain conjugated to the N-terminus of GGGAAAKRK [254], proved useful in promoting self-assembly of peptides to distinct nanocarriers with a hydrophobic core. Accordingly, surfactant-like palmitoyl-GGGAAAKRK formed peptide nanofibers (PNFs) in the presence of siRNA specific for the down-regulation of BCL2 protein. Human SH-SY5Y cells showed significant uptake of PNF:siBCL2 constructs in vitro and silencing of *BCL2* in specific loci of rat brains demonstrated effective delivery of siRNA. In another example, a branched amphiphilic peptide comprising oligolysine segments with DNA binding properties formed different structures depending on the peptide/DNA ratio: at high peptide/DNA ratio, it coated the DNA surface forming nanofibers and at low peptide/DNA ratio, it condensed the DNA into nanometer-sized compacted structures [255]. Using pDNA encoding green fluorescent protein (GFP) as cargo, the peptide nanocarrier demonstrated higher transfection efficiency in HeLa cells compared to Lipofectin (commercial transfection agent) when the total number of transfectants alive was considered.

Another type of versatile and reproducible supramolecular nanocarrier with well-defined structure and composition are dendrimers [256]. Many types of dendrimers including peptide dendrimers (PPI; [257]), poly(L-lysine) dendrimers, and polyamidoamine (PAMAM) dendrimers that display electrostatic interactions with nucleic acids and protect the cargo from degradation, are particularly suited for gene delivery. In addition, dendrimers lend themselves to surface conjugation of peptide moieties that enhance gene delivery. Conjugation of TAT (HIV transactivator of transcription) peptide to PAMAM dendrimer formed nanometer-sized (105 nm–115 nm) ‘dendriplexes’ with GFP pDNA [258] that displayed an increased transfection efficiency in Vero cells compared to PAMAM without TAT.

### Stimuli-Responsive Gene Delivery Systems

Peptide-based assemblies are particularly attractive for developing stimuli-responsive therapeutic nanocarriers for several reasons: they are biocompatible, readily degraded and then removed from the organism, but most importantly, highly sensitive to environmental conditions. Small changes in external factors, such as temperature or pH, can induce transformation of secondary structures (α-helices, β-sheets, and β-turns), thereby affecting the morphology and concomitantly the function and bio-activity of the polypeptides [259]. Moreover, control over cargo release is a highly sought-after feature in gene delivery systems and stimuli-responsive nanocarriers can deliver nucleic acids more efficiently by reducing unspecific release. To date, peptide vectors forming complexes with DNA by electrostatic interactions still make up the majority of peptide-based nanocarriers. As stimuli-responsiveness can be readily obtained by modifying the peptide sequence, these nanoparticles (peptiplexes) represent the main targets for a control of cargo release by external stimuli. However, nano-assemblies, based on their modularity offer not only increased DNA loading capacity but are also more susceptible towards environmental stimuli.

The pH-responsive peptides have been intensively investigated in delivery and diagnostic systems because pH variations are typical for many biological systems, intracellular compartments (lysosomes and endosomes), specific organs (gastrointestinal tract and vagina), and pathological conditions [260,261]. Particularly, the microenvironment of many tumor tissues has a lower pH (<6.5) compared to normal tissues (pH 7.4). Thus, polypeptides that change conformation in a pH-dependent fashion can find application in selective binding to cancer sites which can be exploited for tumor diagnosis and treatment. Nanocarriers taken up by endocytosis encounter acidification in the endosome, which is exploited by pH-responsive peptides to increase endosomal escape. For example, (Fmoc)2KH7-TAT, an amphiphilic, pH-responsive chimeric peptide [262], complexed with pGL-3 reporter plasmid mediated transfection of 293T and HeLa cells by promoting endosomal escape via protonation of KH residues. Moreover, co-delivery of p53 plasmid and doxorubicin using (Fmoc)2KH7-TAT self-assembled micelleplexes inhibited cell growth in vitro and tumor growth in vivo.

Nanocarriers with peptide-mediated redox-sensitivity have emerged as a fascinating type of biomedical material with potential for triggered gene and drug delivery inside cells. Sensitivity of peptides to the redox state is provided by disulphide bonds, diselenide bonds, succinimide-thioether linkage or by redox sensitive groups, such as ‘‘trimethyl-locked’’ benzoquinone [263]. In a reducing environment, redox-responsive nanocarriers undergo a change in conformation and release their cargo. A major advantage of redox-responsive nanocarriers is their stability in normal tissues which avoids cytotoxicity caused by the unwanted release of therapeutic cargo. Tumor tissues show 4-fold higher glutathione levels compared to healthy tissue and thus triggers redox-responsive cargo release [264].

A “smart”, redox-sensitive peptide designed to trigger the assembly of gadolinium nanoparticles inside cells, was successfully applied in magnetic resonance imaging of tumors in a xenograft mouse model [265]. Acetyl-RVRR-C(StBu)-K(Gd-DOTA)-CBT contains an RVRR sequence which mediates cell membrane translocation but is also a cleavage site for intracellular furin, typically upregulated in many tumors, and a disulphided Cys motif. After entering the cell, the disulfide bond is reduced by intracellular glutathione (GSH) and subsequently, the RVRR motif is cleaved by furin in situ. The cleavage product quickly condenses to amphiphilic dimers that self-assemble via π-π stacking into Gd-containing nanoparticles.

**Figure 9:**
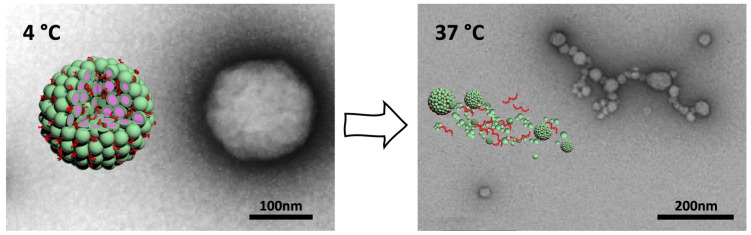
Schematic representation and TEM micrograph of the self-assembled (HR)3gT multi-compartment micellar nano-assembly (MCM) at 4 °C (*left*) and temperature-induced disassembly of MCMs into disperse or clustered smaller MCMs and individual micelles at 37 °C (*right*). Modified from [20] with permission from the Royal Society of Chemistry.

Peptide structures and the weak interactions contributing to self-assembly of peptide-based nanocarriers are inherently sensitive to temperature [266,267]. An interesting example of a thermo-responsive, purely peptidic DNA nanocarrier are multi-compartment micellar nanoparticles (MCM-NPs) assembled from (HR)3gT peptide (Figure 9) [20].

Although the multicompartment micellar structure of NPs was stable at 4 °C, increasing the temperature to 37 °C triggered structural changes that led to the disassembly into smaller MCMs and individual micelles after several hours. On account of the high cellular uptake efficiency and thermo-responsive disassembly at physiological temperature, MCM-NPs are a promising DNA delivery vehicle with great potential for application in vivo. Similar multicompartment micellar NPs assembled from H_3_SSgT peptide bearing a disulfide functional group between hydrophilic and hydrophobic domain, were developed for redox-responsive codelivery of oligonucleotides and drugs [268]. The disulfide bond conferred responsiveness to physiological concentrations of reducing agent upon NPs, resulting in release of the incorporated cargo. The advantage of a supramolecular multicompartment structure over individual micelles lies in the increased capacity for oligonucleotide condensation [269]. Together with the ability to entrap various hydrophobic cargos, this makes MCM-NPs well-suited for biomedical applications.

Light has received much attention as an external stimulus, as it provides spatiotemporal control that can be triggered remotely. By crosslinking peptides with specific light-absorbing molecules it is possible to obtain photo-responsive conjugates that allow for light-stimulated assembly of nanostructures or light-induced release of cargo molecules. Such light-sensitive conjugates of peptides and photosensitizers can serve as light-controllable phototherapeutic agents [270].

Photo-crosslinking by UV (254 nm) of poly(ethylene glycol)-*b*-poly(l-glutamic acid) diblock copolymer was shown to convert the core of self-assembled core-shell micellar structures to nanogels that, depending on the composition of the copolymer, could release drug payload in a pH-dependent manner [271]. These results indicated the potential of nanogels fabricated by photo-crosslinking of polypeptide micelles as intelligent delivery systems.

Micelles with a photocleavable poly(S-(o-nitrobenzyl)-l-cysteine) (PNBC) core surrounded by a hydrophilic poly(ethylene glycol) (PEO) corona were also obtained by self-assembly of PNBC-b-PEO amphiphilic block copolymer [272]. UV irradiation (365 nm) of these micelles gradually removed nitrobenzyl groups from PNBC-b-PEO resulting in a shrinkage of the micelles. If micelles were prepared in the presence of doxorubicin, a photo-triggered release of the drug was observed in vitro. Since self-assembly is achieved in aqueous solution, photocleavable polypeptide-based block copolymers lend themselves to developing photoresponsive nanomedicines for anticancer therapy.

## 4. Combinatorial Approach for Advanced Nucleic Acid Delivery

In view of gene therapy, endowing nanocarries with targeting features and stimuli-responsiveness that provides site-specific, triggerable control over cargo release could optimize delivery efficacy, and, at the same time, minimize adverse effects.

**Figure 10:**
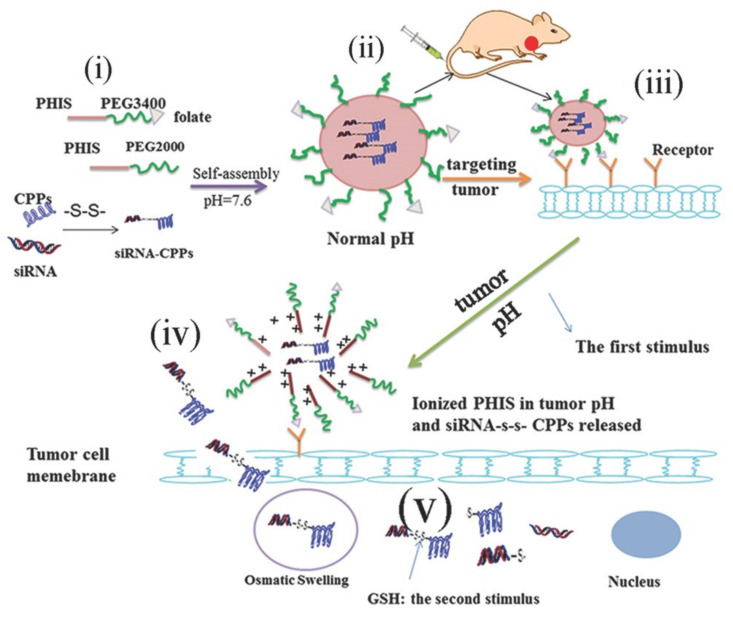
Diagram of F-ASPM formation and the mechanism of siRNA delivery into cancer cells. (**i**) synthesis of PEG-PHIS and F-PEG-PHIS. (**ii**) self-assembly of amphiphilic block copolymers in the presence of siRNA-CPP into acid-sensitive nanocarriers with active targeting ability (F-ASPM). (**iii**) binding of F-ASPM to cancer cells by folate receptor targeting. (**iv**) pH-stimulated release of siRNA-CPPs from F-ASPM. (**v**) GSH-mediated cleavage of disulfide bond of siRNA-CPPs leading to release free siRNA into cytosol. Reprinted with permission from [273]. Copyright 2016 John Wiley and Sons.

For example, folate-receptor targeting, acid-sensitive polymeric micelles (F-ASPM) have been successfully applied to deliver siRNA to breast cancer cells (Figure 10) [273]. In this approach, poly (L-histidine)-polythelene-glycol (PEG-PHIS) and folate-conjugated PEG-PHIS amphiphilic block copolymers in the presence of CPP-coupled siRNA self-assembled into micelles where folate functioned as targeting ligand and histidine residues provided pH-responsiveness. As PEG-PHIS block copolymers have a pKb of 6.5–7.0, micelles formed by this copolymer dissociate at pH 6.5–7.0 which renders them suitable for constructing drug/DNA delivery systems that are sensitive to the extracellular tumor environment. In addition, c-myc silencing siRNA conjugation to a CPP was obtained by reduction-sensitive disulfide bonding which turned the resulting micelles into a dual responsive nanocarrier able to target tumor cells where release and delivery of siRNA are promoted by the respective peptide sequences.

The combination of targeting and stimuli-responsiveness is also provided by ROSE, a redox-sensitive, oligopeptide-guided, self-assembling, and efficiency-enhanced carrier system [274]. In ROSE, adamantyl-PEG chains with and without disulfide bonded SP94 targeting oligopeptide, mixed with hydroxypropyl-β-cyclodextrin formed supramolecular complexes condensing tumor-suppressor microRNA-34a (miR-34a). Oligopeptide-guided specificity for hepatocarcinoma cells and release of miRNA following disulfide cleavage in the reducing environment significantly improved the tumor-suppressing effect of ROSE/miR-34a over conventional gene delivery strategies.

## 5. Conclusions

Since the introduction of peptides as potential delivery system for a variety of therapeutic cargos, extensive research has focused on their application in gene therapy. To be suitably tailored for gene therapy, peptide-based nanocarriers must comply with issues of targeting, cellular uptake, and intracellular trafficking, all of which involve biological membranes and how they can be overcome. A combinatorial approach, e.g., designer peptides composed of cationic cell-penetrating and hydrophobic endosomal escape domains in combination with a gene carrier peptide composed of targeting and cationic DNA-binding domains affording triggered, site-specific (cytosol, nucleus, mitochondria) release of nucleic acids, may offer some improvement of efficacy. Other properties, including, but not limited to, low cytotoxicity, target specificity, biodegradability, and cost and time efficiency of synthesis greatly contribute to the potential of peptides in nanomedicine. Nevertheless, to broadly realize bench to bedside translation of peptide-related gene delivery systems, innovative technologies need to be pursued to achieve peptide-based nanocarriers that more specifically and efficiently deliver nucleic acids or nucleic acid modifying systems to the desired sites. In many cases, such nanocarriers would further benefit from either sustained or triggered delivery options.

Advances in peptide development have made peptide-assisted gene delivery more efficient in vitro and, in some instances, in small animal models [275]. For example, cell and tissue selectivity could be greatly enhanced in the newest generation of CPPs [276]. Other advances which allow for improved performance with regard to targeting and delivery of nucleic acids include adapting peptide sequences to facilitate escape or release from intracellular vesicles or respond to environmental stimuli for a controlled release of cargo, and the development of composite, multivalent peptide-based, or peptide-coupled structures.

Intriguingly, while revolutionary and versatile peptide tools have inspired a great deal of hope regarding the treatment of genetic diseases, peptide nanocarriers are awaiting clinical translation. For example, none of the nanocarriers associated with CPPs have so far been approved for clinical studies. Evidently, besides overcoming membrane barriers, a string of challenges remain that need to be tackled for peptide nanocarriers to make a breakthrough in clinical application whereas lipid-based formulations, for all their drawbacks [277], are being used in the field of gene therapy and as a delivery vehicle in mRNA-based vaccines [278]. Short circulation half-lives, inadequate biodistribution, and poor chemical and physical serum stability, especially susceptibility to proteolytic degradation associated with off-target nucleic acid release, hamper clinical translation of peptide-based nanocarriers. Refining their preparation with regard to gene loading efficiency and product homogeneity would be a possible improvement. However, reaching the final target with high selectivity and adequate accumulation at the target site remains a major issue for in vivo applications. Here, peptide modifications (unnatural amino acids, cyclization) and conjugate molecules (PEGylation, hydrocarbon chains) that prolong the circulation time and enhance the structural stability of nanocarriers in the serum come to mind [279,280]. Peptidomimetics, often based on natural peptide sequences, that exhibit improved proteolytic stability or even new folds and morphologies designed to enhance bio availability, improve transport through the blood–brain barrier, or reduce the rate of clearance, are emerging. However, their modifications bear the risk of reducing potency or even introducing toxicity, for example D-amino acids [281]. Another alternative to clear abovementioned hurdles is developing advanced multifunctional carriers comprising various agents, each of which can overcome the barrier through distinct dictated functions. Undoubtedly, peptides offer the largest potential when it comes to nucleic acid condensation, targeting, endosomal escape, and subcellular localization as a part of multifunctional advanced delivery systems. But again, the combination of functionalities bears the danger of affecting the individual functions. Thus, extensive research on the development of stimuli-responsive purely peptidic systems with suitable physicochemical properties for nucleic acid delivery is being pursued at many levels.

A better understanding of the mechanisms by which peptide-based delivery systems use to overcome membrane but also other biological barriers, together with advancements in the synthesis of innovative materials tailored to environmental conditions and extensive in vivo studies herald a bright future for peptide-based delivery systems in gene therapy and in nanomedicine in general.

## Figures and Tables

**Table 1:** Examples of peptides used for targeting in cancer gene therapy.

Peptide Name	Cargo	Cancer Type	Ref.
RGD	siRNA	breast	[35]
cRGD	siRNA	brain	[36]
	siRNA	skin	[37]
iRGD	siRNA	pancreatic	[38]
	siRNA	lung	[39]
RGDfC	siRNA and doxorubicin	liver	[40]
CRGDK	siRNA and BAplatin	breast	[41]
CGKRK	siRNA	breast and brain	[42]
KTLLPTP	siRNA and paclitaxel	pancreatic	[43]
HAIYPRH	siRNA and doxorubicin	breast	[44]
LyP-1 and iRGD	siRNA	ovarian	[45]
YHWYGYTPQNVI	siRNA	liver	[46]
T7	pDNA	bone	[47]

**Table 2:** CPP classification based on physicochemical properties.

Cell-Penetrating Peptides								
Cationic			Amphipathic			Hydrophobic		
Name(origin)	Cargo	Ref.	Name(origin)	Cargo	Ref.	Name(origin)	Cargo	Ref.
Diatos Peptide Vectors (DPV)	siRNA	[60]	MPG	pDNA siRNA	[61,62]	C105Y	pDNA	[63,64,65,66]
HIV-1 twinarginine translocation (TAT)	pDNA siRNA	[67,68,69,70,71,72,73,74]	Transportan	pDNA siRNA	[72,75,76]	K-FGF	pDNA	[77]
arginine-rich peptides	pDNA siRNA	[78,79,80,81,82]	NickFect (NF)	pDNA siRNA	[83,84,85,86]	Bip	pDNA	[87]
Polyarginine	pDNA	[75,76,88,89,90]	PepFect (PF)	pDNA mRNA	[91,92]	Melittin-derived peptides	siRNA	[93]
Penetratin	pDNA	[94,95,96]	MAP	siRNA	[97]			
L5a	pDNA	[98,99]	Crotamine	pDNA	[100,101,102]			
			VP22	pDNA	[103,104]			
Protamine	pDNA mRNA	[105,106,107,108,109]	Antennapedia (Antp)	AON siRNA	[110,111]			
			Pep-1	pDNA	[112,113]			
			CADY	siRNA	[114,115,116]			
			FGF	pDNA	[77]			
			pVEC	pDNA	[117,118]			

## Data Availability

Not applicable.

## References

[1] Gillmore J.D., Gane E., Taubel J., Kao J., Fontana M., Maitland M.L., Seitzer J., O’Connell D., Walsh K.R., Wood K. (2021). CRISPR-Cas9 In Vivo Gene Editing for Transthyretin Amyloidosis. N. Engl. J. Med..

[2] Rosenblum D., Gutkin A., Kedmi R., Ramishetti S., Veiga N., Jacobi A.M., Schubert M.S., Friedmann-Morvinski D., Cohen Z.R., Behlke M.A. (2020). CRISPR-Cas9 Genome Editing Using Targeted Lipid Nanoparticles for Cancer Therapy. Sci. Adv..

[3] Pardi N., Hogan M.J., Porter F.W., Weissman D. (2018). MRNA Vaccines—A New Era in Vaccinology. Nat. Rev. Drug Discov..

[4] Jackson L.A., Anderson E.J., Rouphael N.G., Roberts P.C., Makhene M., Coler R.N., McCullough M.P., Chappell J.D., Denison M.R., Stevens L.J. (2020). An MRNA Vaccine against SARS-CoV-2 - Preliminary Report. N. Engl. J. Med..

[5] Fogel D.B. (2018). Factors Associated with Clinical Trials That Fail and Opportunities for Improving the Likelihood of Success: A Review. Contemp. Clin. Trials Commun..

[6] Seyhan A.A. (2019). Lost in Translation: The Valley of Death across Preclinical and Clinical Divide—Identification of Problems and Overcoming Obstacles. Transl. Med. Commun..

[7] Blanco E., Shen H., Ferrari M. (2015). Principles of Nanoparticle Design for Overcoming Biological Barriers to Drug Delivery. Nat. Biotechnol..

[8] Muttenthaler M., King G.F., Adams D.J., Alewood P.F. (2021). Trends in Peptide Drug Discovery. Nat. Rev. Drug Discov..

[9] Kumari S., Mg S., Mayor S. (2010). Endocytosis Unplugged: Multiple Ways to Enter the Cell. Cell Res..

[10] Parodi A., Corbo C., Cevenini A., Molinaro R., Palomba R., Pandolfi L., Agostini M., Salvatore F., Tasciotti E. (2015). Enabling Cytoplasmic Delivery and Organelle Targeting by Surface Modification of Nanocarriers. Nanomedicine.

[11] Petros R.A., DeSimone J.M. (2010). Strategies in the Design of Nanoparticles for Therapeutic Applications. Nat. Rev. Drug Discov..

[12] Ruoslahti E. (2012). Peptides as Targeting Elements and Tissue Penetration Devices for Nanoparticles. Adv. Mater..

[13] Jeong W., Bu J., Kubiatowicz L.J., Chen S.S., Kim Y., Hong S. (2018). Peptide–Nanoparticle Conjugates: A next Generation of Diagnostic and Therapeutic Platforms?. Nano Converg..

[14] Hossen S., Hossain M.K., Basher M.K., Mia M.N.H., Rahman M.T., Uddin M.J. (2019). Smart Nanocarrier-Based Drug Delivery Systems for Cancer Therapy and Toxicity Studies: A Review. J. Adv. Res..

[15] Lee A.C.-L., Harris J.L., Khanna K.K., Hong J.-H. (2019). A Comprehensive Review on Current Advances in Peptide Drug Development and Design. Int. J. Mol. Sci..

[16] Cooper B.M., Iegre J., Donovan D.H.O., Halvarsson M.Ö., Spring D.R. (2021). Peptides as a Platform for Targeted Therapeutics for Cancer: Peptide–Drug Conjugates (PDCs). Chem. Soc. Rev..

[17] Mitchell M.J., Billingsley M.M., Haley R.M., Wechsler M.E., Peppas N.A., Langer R. (2021). Engineering Precision Nanoparticles for Drug Delivery. Nat. Rev. Drug Discov..

[18] Sandra F., Khaliq N.U., Sunna A., Care A. (2019). Developing Protein-Based Nanoparticles as Versatile Delivery Systems for Cancer Therapy and Imaging. Nanomaterials.

[19] Avci F.G., Sariyar Akbulut B., Ozkirimli E. (2018). Membrane Active Peptides and Their Biophysical Characterization. Biomolecules.

[20] Tarvirdipour S., Schoenenberger C.-A., Benenson Y., Palivan C.G. (2020). A Self-Assembling Amphiphilic Peptide Nanoparticle for the Efficient Entrapment of DNA Cargoes up to 100 Nucleotides in Length. Soft Matter..

[21] Sánchez-Navarro M., Teixidó M., Giralt E. (2017). Jumping Hurdles: Peptides Able To Overcome Biological Barriers. Acc. Chem. Res..

[22] Cabral B.P., da Graça Derengowski Fonseca M., Mota F.B. (2018). The Recent Landscape of Cancer Research Worldwide: A Bibliometric and Network Analysis. Oncotarget.

[23] Ruoslahti E. (2002). Specialization of Tumour Vasculature. Nat. Rev. Cancer.

[24] Marelli U.K., Rechenmacher F., Sobahi T.R.A., Mas-Moruno C., Kessler H. (2013). Tumor Targeting via Integrin Ligands. Front. Oncol..

[25] Brown K.C. (2010). Peptidic Tumor Targeting Agents: The Road from Phage Display Peptide Selections to Clinical Applications. Curr. Pharm. Des..

[26] Zhao N., Qin Y., Liu H., Cheng Z. (2018). Tumor-Targeting Peptides: Ligands for Molecular Imaging and Therapy. Anticancer Agents Med. Chem..

[27] Li Z.J., Cho C.H. (2012). Peptides as Targeting Probes against Tumor Vasculature for Diagnosis and Drug Delivery. J. Transl. Med..

[28] Ko J.K., Auyeung K.K. (2014). Identification of Functional Peptides from Natural and Synthetic Products on Their Anticancer Activities by Tumor Targeting. Curr. Med. Chem..

[29] Zhao J., Santino F., Giacomini D., Gentilucci L. (2020). Integrin-Targeting Peptides for the Design of Functional Cell-Responsive Biomaterials. Biomedicines.

[30] Wu P.-H., Opadele A.E., Onodera Y., Nam J.-M. (2019). Targeting Integrins in Cancer Nanomedicine: Applications in Cancer Diagnosis and Therapy. Cancers.

[31] Huang Y., Wang X., Huang W., Cheng Q., Zheng S., Guo S., Cao H., Liang X.-J., Du Q., Liang Z. (2015). Systemic Administration of SiRNA via CRGD-Containing Peptide. Sci. Rep..

[32] Roth L., Agemy L., Kotamraju V.R., Braun G., Teesalu T., Sugahara K.N., Hamzah J., Ruoslahti E. (2012). Transtumoral Targeting Enabled by a Novel Neuropilin-Binding Peptide. Oncogene.

[33] Jiang Y., Liu S., Zhang Y., Li H., He H., Dai J., Jiang T., Ji W., Geng D., Elzatahry A.A. (2017). Magnetic Mesoporous Nanospheres Anchored with LyP-1 as an Efficient Pancreatic Cancer Probe. Biomaterials.

[34] Song N., Zhao L., Zhu M., Zhao J. (2019). Recent Progress in LyP-1-Based Strategies for Targeted Imaging and Therapy. Drug Deliv..

[35] Vaidya A.M., Sun Z., Ayat N., Schilb A., Liu X., Jiang H., Sun D., Scheidt J., Qian V., He S. (2019). Systemic Delivery of Tumor-Targeting SiRNA Nanoparticles against an Oncogenic LncRNA Facilitates Effective Triple-Negative Breast Cancer Therapy. Bioconjug. Chem..

[36] He S., Cen B., Liao L., Wang Z., Qin Y., Wu Z., Liao W., Zhang Z., Ji A. (2017). A Tumor-Targeting CRGD-EGFR SiRNA Conjugate and Its Anti-Tumor Effect on Glioblastoma in Vitro and in Vivo. Drug Deliv..

[37] Zhang Y., Li S., Zhou X., Sun J., Fan X., Guan Z., Zhang L., Yang Z. (2019). Construction of a Targeting Nanoparticle of 3′,3″-Bis-Peptide-SiRNA Conjugate/Mixed Lipid with Postinserted DSPE-PEG2000-CRGD. Mol. Pharm..

[38] Lo J.H., Hao L., Muzumdar M.D., Raghavan S., Kwon E.J., Pulver E.M., Hsu F., Aguirre A.J., Wolpin B.M., Fuchs C.S. (2018). IRGD-Guided Tumor-Penetrating Nanocomplexes for Therapeutic SiRNA Delivery to Pancreatic Cancer. Mol. Cancer Ther..

[39] Zhou Y., Yuan Y., Liu M., Hu X., Quan Y., Chen X. (2019). Tumor-Specific Delivery of KRAS SiRNA with IRGD-Exosomes Efficiently Inhibits Tumor Growth. ExRNA.

[40] Xia Y., Xu T., Wang C., Li Y., Lin Z., Zhao M., Zhu B. (2017). Novel Functionalized Nanoparticles for Tumor-Targeting Co-Delivery of Doxorubicin and SiRNA to Enhance Cancer Therapy. Int. J. Nanomed..

[41] Bai Y., Li Z., Liu L., Sun T., Fan X., Wang T., Gou Z., Tan S. (2019). Tumor-Targeting Peptide for Redox-Responsive Pt Prodrug and Gene Codelivery and Synergistic Cancer Chemotherapy. ACS Appl. Bio Mater..

[42] Sharma M., El-Sayed N.S., Do H., Parang K., Tiwari R.K., Aliabadi H.M. (2017). Tumor-Targeted Delivery of SiRNA Using Fatty Acyl-CGKRK Peptide Conjugates. Sci. Rep..

[43] Li Y., Wang H., Wang K., Hu Q., Yao Q., Shen Y., Yu G., Tang G. (2017). Targeted Co-Delivery of PTX and TR3 SiRNA by PTP Peptide Modified Dendrimer for the Treatment of Pancreatic Cancer. Small.

[44] Wan W., Qu C., Zhou Y., Zhang L., Chen M., Liu Y., You B., Li F., Wang D., Zhang X. (2019). Doxorubicin and SiRNA-PD-L1 Co-Delivery with T7 Modified ROS-Sensitive Nanoparticles for Tumor Chemoimmunotherapy. Int. J. Pharm..

[45] Kim B., Sun S., Varner J.A., Howell S.B., Ruoslahti E., Sailor M.J. (2019). Securing the Payload, Finding the Cell, and Avoiding the Endosome: Peptide-Targeted, Fusogenic Porous Silicon Nanoparticles for Delivery of SiRNA. Adv. Mater..

[46] Liang Y., Peng J., Li N., Yu-Wai-Man C., Wang Q., Xu Y., Wang H., Tagalakis A.D., Du Z. (2019). Smart Nanoparticles Assembled by Endogenous Molecules for SiRNA Delivery and Cancer Therapy via CD44 and EGFR Dual-Targeting. Nanomed. Nanotechnol. Biol. Med..

[47] Lu Y., Jiang W., Wu X., Huang S., Huang Z., Shi Y., Dai Q., Chen J., Ren F., Gao S. (2018). Peptide T7-Modified Polypeptide with Disulfide Bonds for Targeted Delivery of Plasmid DNA for Gene Therapy of Prostate Cancer. Int. J. Nanomed..

[48] Desale K., Kuche K., Jain S. (2021). Cell-Penetrating Peptides (CPPs): An Overview of Applications for Improving the Potential of Nanotherapeutics. Biomater. Sci..

[49] Torchilin V.P. (2008). Cell Penetrating Peptide-Modified Pharmaceutical Nanocarriers for Intracellular Drug and Gene Delivery. Pept. Sci..

[50] Wang F., Wang Y., Zhang X., Zhang W., Guo S., Jin F. (2014). Recent Progress of Cell-Penetrating Peptides as New Carriers for Intracellular Cargo Delivery. J. Control. Release.

[51] Khan M.M., Filipczak N., Torchilin V.P. (2021). Cell Penetrating Peptides: A Versatile Vector for Co-Delivery of Drug and Genes in Cancer. J. Control. Release.

[52] Tian Y., Zhou M., Shi H., Gao S., Xie G., Zhu M., Wu M., Chen J., Niu Z. (2018). Integration of Cell-Penetrating Peptides with Rod-like Bionanoparticles: Virus-Inspired Gene-Silencing Technology. Nano Lett..

[53] Conde J., Ambrosone A., Hernandez Y., Tian F., McCully M., Berry C.C., Baptista P.V., Tortiglione C., de la Fuente J.M. (2015). 15 Years on SiRNA Delivery: Beyond the State-of-the-Art on Inorganic Nanoparticles for RNAi Therapeutics. Nano Today.

[54] Kang Z., Meng Q., Liu K. (2019). Peptide-Based Gene Delivery Vectors. J. Mater. Chem. B.

[55] Madani F., Lindberg S., Langel Ü., Futaki S., Gräslund A. (2011). Mechanisms of Cellular Uptake of Cell-Penetrating Peptides. J. Biophys..

[56] Lindgren M., Langel Ü., Langel Ü. (2011). Classes and Prediction of Cell-Penetrating Peptides. Cell-Penetrating Peptides: Methods and Protocols.

[57] Xie J., Bi Y., Zhang H., Dong S., Teng L., Lee R.J., Yang Z. (2020). Cell-Penetrating Peptides in Diagnosis and Treatment of Human Diseases: From Preclinical Research to Clinical Application. Front. Pharmacol..

[58] Trabulo S., Cardoso A.L., Mano M., De Lima M.C.P. (2010). Cell-Penetrating Peptides—Mechanisms of Cellular Uptake and Generation of Delivery Systems. Pharmaceuticals.

[59] Derakhshankhah H., Jafari S. (2018). Cell Penetrating Peptides: A Concise Review with Emphasis on Biomedical Applications. Biomed. Pharmacother..

[60] Alluis B., Fruchart J.-S. (2013). Cell Penetrating Peptide Conjugates for Delivering of Nucleic Acids into a Cell 2013.

[61] Simeoni F., Morris M.C., Heitz F., Divita G. (2003). Insight into the Mechanism of the Peptide-based Gene Delivery System MPG: Implications for Delivery of SiRNA into Mammalian Cells. Nucleic Acids Res..

[62] Crombez L., Morris M.C., Dufort S., Aldrian-Herrada G., Nguyen Q., Mc Master G., Coll J.-L., Heitz F., Divita G. (2009). Targeting Cyclin B1 through Peptide-Based Delivery of SiRNA Prevents Tumour Growth. Nucleic Acids Res..

[63] Rhee M., Davis P. (2006). Mechanism of Uptake of C105Y, a Novel Cell-Penetrating Peptide. J. Biol. Chem..

[64] Sun W., Ziady A.G., Foote R.S., Lee J.W. (2009). Real-Time Imaging of Gene Delivery and Expression with DNA Nanoparticle Technologies. Micro and Nano Technologies in Bioanalysis: Methods and Protocols.

[65] Saamoah-Moffatt S., Wiehle S., Christina R.J. (2004). Enhanced Gene Delivery by a Novel Tumor-Specific Vector Containing Peptide and Nucleic Acid Based Nuclear Translocation Signals. Mol. Ther..

[66] Lee M., Choi J.S., Ko K.S. (2005). 202. DNA Delivery to the Mitochondria Sites Using Leader Peptide Conjugated Polyethylenimine. Mol. Ther..

[67] Rajala A., Wang Y., Zhu Y., Ranjo-Bishop M., Ma J.-X., Mao C., Rajala R.V.S. (2014). Nanoparticle-Assisted Targeted Delivery of Eye-Specific Genes to Eyes Significantly Improves the Vision of Blind Mice In Vivo. Nano Lett..

[68] Wu Z., Zhan S., Fan W., Ding X., Wu X., Zhang W., Fu Y., Huang Y., Huang X., Chen R. (2016). Peptide-Mediated Tumor Targeting by a Degradable Nano Gene Delivery Vector Based on Pluronic-Modified Polyethylenimine. Nanoscale Res. Lett..

[69] Dong S., Zhou X., Yang J. (2016). TAT Modified and Lipid–PEI Hybrid Nanoparticles for Co-Delivery of Docetaxel and PDNA. Biomed. Pharmacother..

[70] Ishiguro S., Alhakamy N.A., Uppalapati D., Delzeit J., Berkland C.J., Tamura M. (2017). Combined Local Pulmonary and Systemic Delivery of AT2R Gene by Modified TAT Peptide Nanoparticles Attenuates Both Murine and Human Lung Carcinoma Xenografts in Mice. J. Pharm. Sci..

[71] Bahadoran A., Ebrahimi M., Yeap S.K., Safi N., Moeini H., Hair-Bejo M., Hussein M.Z., Omar A.R. (2017). Induction of a Robust Immune Response against Avian Influenza Virus Following Transdermal Inoculation with H5-DNA Vaccine Formulated in Modified Dendrimer-Based Delivery System in Mouse Model. Int. J. Nanomed..

[72] Li L., Hu S., Chen X. (2018). Non-Viral Delivery Systems for CRISPR/Cas9-Based Genome Editing: Challenges and Opportunities. Biomaterials.

[73] Koizumi K., Nakamura H., Iijima M., Matsuzaki T., Somiya M., Kumasawa K., Kimura T., Kuroda S. (2019). In Vivo Uterine Local Gene Delivery System Using TAT-Displaying Bionanocapsules. J. Gene Med..

[74] Yi A., Sim D., Lee Y.-J., Sarangthem V., Park R.-W. (2020). Development of Elastin-like Polypeptide for Targeted Specific Gene Delivery in Vivo. J. Nanobiotechnol..

[75] Yoon J.Y., Yang K.-J., Park S.-N., Kim D.-K., Kim J.-D. (2016). The Effect of Dexamethasone/Cell-Penetrating Peptide Nanoparticles on Gene Delivery for Inner Ear Therapy. Int. J. Nanomed..

[76] Khalil I.A., Harashima H. (2018). An Efficient PEGylated Gene Delivery System with Improved Targeting: Synergism between Octaarginine and a Fusogenic Peptide. Int. J. Pharm..

[77] Lee J., Jung J., Kim Y.-J., Lee E., Choi J.S. (2014). Gene Delivery of PAMAM Dendrimer Conjugated with the Nuclear Localization Signal Peptide Originated from Fibroblast Growth Factor 3. Int. J. Pharm..

[78] Li Y., Li Y., Wang X., Lee R.J., Teng L. (2015). Fatty Acid Modified Octa-Arginine for Delivery of SiRNA. Int. J. Pharm..

[79] Van Rossenberg S., van Keulen A., Drijfhout J.-W., Vasto S., Koerten H.K., Spies F., van ’t Noordende J., van Berkel T., Biessen E.a.L. (2004). Stable Polyplexes Based on Arginine-Containing Oligopeptides for in Vivo Gene Delivery. Gene Ther..

[80] Br L., Md L., Hj C., Hj L. (2012). Arginine-Rich Cell-Penetrating Peptides Deliver Gene into Living Human Cells. Gene.

[81] Liu C., Liu X., Rocchi P., Qu F., Iovanna J.L., Peng L. (2014). Arginine-Terminated Generation 4 PAMAM Dendrimer as an Effective Nanovector for Functional SiRNA Delivery in Vitro and in Vivo. Bioconjug. Chem..

[82] Kato T., Yamashita H., Misawa T., Nishida K., Kurihara M., Tanaka M., Demizu Y., Oba M. (2016). Plasmid DNA Delivery by Arginine-Rich Cell-Penetrating Peptides Containing Unnatural Amino Acids. Bioorg. Med. Chem..

[83] Arukuusk P., Pärnaste L., Hällbrink M., Langel Ü., Langel Ü. (2015). PepFects and NickFects for the Intracellular Delivery of Nucleic Acids. Cell-Penetrating Peptides: Methods and Protocols.

[84] Margus H., Arukuusk P., Langel Ü., Pooga M. (2016). Characteristics of Cell-Penetrating Peptide/Nucleic Acid Nanoparticles. Mol. Pharm..

[85] Freimann K., Arukuusk P., Kurrikoff K., Pärnaste L., Raid R., Piirsoo A., Pooga M., Langel Ü. (2018). Formulation of Stable and Homogeneous Cell-Penetrating Peptide NF55 Nanoparticles for Efficient Gene Delivery In Vivo. Mol. Ther. Nucleic Acids.

[86] Padari K., Porosk L., Arukuusk P., Pooga M., Gissberg O., Zain R., Lundin K.E. (2019). Characterization of Peptide–Oligonucleotide Complexes Using Electron Microscopy, Dynamic Light Scattering, and Protease Resistance Assay. Oligonucleotide-Based Therapies: Methods and Protocols.

[87] Minchin R.F., Yang S. (2010). Endosomal Disruptors in Non-Viral Gene Delivery. Expert Opin. Drug Deliv..

[88] Jiang Q.-Y., Lai L.-H., Shen J., Wang Q.-Q., Xu F.-J., Tang G.-P. (2011). Gene Delivery to Tumor Cells by Cationic Polymeric Nanovectors Coupled to Folic Acid and the Cell-Penetrating Peptide Octaarginine. Biomaterials.

[89] Oba M., Demizu Y., Yamashita H., Kurihara M., Tanaka M. (2015). Plasmid DNA Delivery Using Fluorescein-Labeled Arginine-Rich Peptides. Bioorg. Med. Chem..

[90] Zhang L., Li Z., Sun F., Xu Y., Du Z. (2016). Effect of Inserted Spacer in Hepatic Cell-Penetrating Multifunctional Peptide Component on the DNA Intracellular Delivery of Quaternary Complexes Based on Modular Design. Int. J. Nanomed..

[91] Van den Brand D., Gorris M.A.J., van Asbeck A.H., Palmen E., Ebisch I., Dolstra H., Hällbrink M., Massuger L.F.A.G., Brock R. (2019). Peptide-Mediated Delivery of Therapeutic MRNA in Ovarian Cancer. Eur. J. Pharm. Biopharm..

[92] Kurrikoff K., Veiman K.-L., Künnapuu K., Peets E.M., Lehto T., Pärnaste L., Arukuusk P., Langel Ü. (2017). Effective in Vivo Gene Delivery with Reduced Toxicity, Achieved by Charge and Fatty Acid -Modified Cell Penetrating Peptide. Sci. Rep..

[93] Hou K.K., Pan H., Lanza G.M., Wickline S.A. (2013). Melittin Derived Peptides for Nanoparticle Based SiRNA Transfection. Biomaterials.

[94] Christiaens B., Dubruel P., Grooten J., Goethals M., Vandekerckhove J., Schacht E., Rosseneu M. (2005). Enhancement of Polymethacrylate-Mediated Gene Delivery by Penetratin. Eur. J. Pharm. Sci..

[95] Zorko M., Langel Ü. (2005). Cell-Penetrating Peptides: Mechanism and Kinetics of Cargo Delivery. Adv. Drug Deliv. Rev..

[96] Liu C., Jiang K., Tai L., Liu Y., Wei G., Lu W., Pan W. (2016). Facile Noninvasive Retinal Gene Delivery Enabled by Penetratin. ACS Appl. Mater. Interfaces.

[97] Mo R.H., Zaro J.L., Shen W.-C. (2012). Comparison of Cationic and Amphipathic Cell Penetrating Peptides for SiRNA Delivery and Efficacy. Mol. Pharm..

[98] Liu B.R., Huang Y.-W., Aronstam R.S., Lee H.-J. (2016). Identification of a Short Cell-Penetrating Peptide from Bovine Lactoferricin for Intracellular Delivery of DNA in Human A549 Cells. PLoS ONE.

[99] Liu B.R., Huang Y.-W., Korivi M., Lo S.-Y., Aronstam R.S., Lee H.-J. (2017). The Primary Mechanism of Cellular Internalization for a Short Cell- Penetrating Peptide as a Nano-Scale Delivery System. Curr. Pharm. Biotechnol..

[100] Freitas V.J.F., Campelo I.S., Silva M.M.A.S., Cavalcanti C.M., Teixeira D.I.A., Camargo L.S.A., Melo L.M., Rádis-Baptista G. (2020). Disulphide-Less Crotamine Is Effective for Formation of DNA–Peptide Complex but Is Unable to Improve Bovine Embryo Transfection. Zygote.

[101] Campeiro J.D., Dam W., Monte G.G., Porta L.C., de Oliveira L.C.G., Nering M.B., Viana G.M., Carapeto F.C., Oliveira E.B., van den Born J. (2019). Long Term Safety of Targeted Internalization of Cell Penetrating Peptide Crotamine into Renal Proximal Tubular Epithelial Cells in Vivo. Sci. Rep..

[102] Nascimento F.D., Hayashi M.A.F., Kerkis A., Oliveira V., Oliveira E.B., Rádis-Baptista G., Nader H.B., Yamane T., dos Santos Tersariol I.L., Kerkis I. (2007). Crotamine Mediates Gene Delivery into Cells through the Binding to Heparan Sulfate Proteoglycans. J. Biol. Chem..

[103] Zavaglia D., Favrot M.-C., Eymin B., Tenaud C., Coll J.-L. (2003). Intercellular Trafficking and Enhanced in Vivo Antitumour Activity of a Non-Virally Delivered P27-VP22 Fusion Protein. Gene Ther..

[104] Chen H.-C.G., Chiou S.-T., Zheng J.-Y., Yang S.-H., Lai S.-S., Kuo T.-Y. (2011). The Nuclear Localization Signal Sequence of Porcine Circovirus Type 2 ORF2 Enhances Intracellular Delivery of Plasmid DNA. Arch. Virol..

[105] Kim N.H., Provoda C., Lee K.-D. (2015). Design and Characterization of Novel Recombinant Listeriolysin O–Protamine Fusion Proteins for Enhanced Gene Delivery. Mol. Pharm..

[106] Rezaee M., Oskuee R.K., Nassirli H., Malaekeh-Nikouei B. (2016). Progress in the Development of Lipopolyplexes as Efficient Non-Viral Gene Delivery Systems. J. Control. Release.

[107] Men K., Zhang R., Zhang X., Huang R., Zhu G., Tong R., Yang L., Wei Y., Duan X. (2018). Delivery of Modified MRNA Encoding Vesicular Stomatitis Virus Matrix Protein for Colon Cancer Gene Therapy. RSC Adv..

[108] Limeres M.J., Suñé-Pou M., Prieto-Sánchez S., Moreno-Castro C., Nusblat A.D., Hernández-Munain C., Castro G.R., Suñé C., Suñé-Negre J.M., Cuestas M.L. (2019). Development and Characterization of an Improved Formulation of Cholesteryl Oleate-Loaded Cationic Solid-Lipid Nanoparticles as an Efficient Non-Viral Gene Delivery System. Colloids Surf. B Biointerfaces.

[109] Zhang F., Li H.-Y., Ferrari E., Soloviev M. (2020). Preparation of Lipid–Peptide–DNA (LPD) Nanoparticles and Their Use for Gene Transfection. Nanoparticles in Biology and Medicine: Methods and Protocols.

[110] Astriab-Fisher A., Sergueev D., Fisher M., Ramsay Shaw B., Juliano R.L. (2002). Conjugates of Antisense Oligonucleotides with the Tat and Antennapedia Cell-Penetrating Peptides: Effects on Cellular Uptake, Binding to Target Sequences, and Biologic Actions. Pharm. Res..

[111] Davidson T.J., Harel S., Arboleda V.A., Prunell G.F., Shelanski M.L., Greene L.A., Troy C.M. (2004). Highly Efficient Small Interfering RNA Delivery to Primary Mammalian Neurons Induces MicroRNA-Like Effects before MRNA Degradation. J. Neurosci..

[112] Muñoz-Morris M.A., Heitz F., Divita G., Morris M.C. (2007). The Peptide Carrier Pep-1 Forms Biologically Efficient Nanoparticle Complexes. Biochem. Biophys. Res. Commun..

[113] Chang J.-C., Liu K.-H., Chuang C.-S., Su H.-L., Wei Y.-H., Kuo S.-J., Liu C.-S. (2013). Treatment of Human Cells Derived from MERRF Syndrome by Peptide-Mediated Mitochondrial Delivery. Cytotherapy.

[114] Crowet J.-M., Lins L., Deshayes S., Divita G., Morris M., Brasseur R., Thomas A. (2013). Modeling of Non-Covalent Complexes of the Cell-Penetrating Peptide CADY and Its SiRNA Cargo. Biochim. Biophys. Acta BBA Biomembr..

[115] Rydström A., Deshayes S., Konate K., Crombez L., Padari K., Boukhaddaoui H., Aldrian G., Pooga M., Divita G. (2011). Direct Translocation as Major Cellular Uptake for CADY Self-Assembling Peptide-Based Nanoparticles. PLoS ONE.

[116] Konate K., Lindberg M.F., Vaissiere A., Jourdan C., Aldrian G., Margeat E., Deshayes S., Boisguerin P. (2016). Optimisation of Vectorisation Property: A Comparative Study for a Secondary Amphipathic Peptide. Int. J. Pharm..

[117] Elmquist A., Lindgren M., Bartfai T., Langel Ü. (2001). VE-Cadherin-Derived Cell-Penetrating Peptide, PVEC, with Carrier Functions. Exp. Cell Res..

[118] Rajpal, Mann A., Khanduri R., Naik R.J., Ganguli M. (2012). Structural Rearrangements and Chemical Modifications in Known Cell Penetrating Peptide Strongly Enhance DNA Delivery Efficiency. J. Control. Release.

[119] Patel S.G., Sayers E.J., He L., Narayan R., Williams T.L., Mills E.M., Allemann R.K., Luk L.Y.P., Jones A.T., Tsai Y.-H. (2019). Cell-Penetrating Peptide Sequence and Modification Dependent Uptake and Subcellular Distribution of Green Florescent Protein in Different Cell Lines. Sci. Rep..

[120] Zhang D., Wang J., Xu D. (2016). Cell-Penetrating Peptides as Noninvasive Transmembrane Vectors for the Development of Novel Multifunctional Drug-Delivery Systems. J. Control. Release.

[121] Gessner I., Neundorf I. (2020). Nanoparticles Modified with Cell-Penetrating Peptides: Conjugation Mechanisms, Physicochemical Properties, and Application in Cancer Diagnosis and Therapy. Int. J. Mol. Sci..

[122] Fischer R., Fotin-Mleczek M., Hufnagel H., Brock R. (2005). Break on through to the Other Side—Biophysics and Cell Biology Shed Light on Cell-Penetrating Peptides. ChemBioChem.

[123] Zhou Y., Han S., Liang Z., Zhao M., Liu G., Wu J. (2020). Progress in Arginine-Based Gene Delivery Systems. J. Mater. Chem. B.

[124] Nam H.Y., Nam K., Hahn H.J., Kim B.H., Lim H.J., Kim H.J., Choi J.S., Park J.-S. (2009). Biodegradable PAMAM Ester for Enhanced Transfection Efficiency with Low Cytotoxicity. Biomaterials.

[125] Tünnemann G., Ter-Avetisyan G., Martin R.M., Stöckl M., Herrmann A., Cardoso M.C. (2008). Live-Cell Analysis of Cell Penetration Ability and Toxicity of Oligo-Arginines. J. Pept. Sci..

[126] Singh T., Murthy A.S.N., Yang H.-J., Im J. (2018). Versatility of Cell-Penetrating Peptides for Intracellular Delivery of SiRNA. Drug Deliv..

[127] Ruseska I., Zimmer A. (2020). Internalization Mechanisms of Cell-Penetrating Peptides. Beilstein J. Nanotechnol..

[128] Guidotti G., Brambilla L., Rossi D. (2017). Cell-Penetrating Peptides: From Basic Research to Clinics. Trends Pharmacol. Sci..

[129] Munyendo W.L., Lv H., Benza-Ingoula H., Baraza L.D., Zhou J. (2012). Cell Penetrating Peptides in the Delivery of Biopharmaceuticals. Biomolecules.

[130] Veiman K.-L., Mäger I., Ezzat K., Margus H., Lehto T., Langel K., Kurrikoff K., Arukuusk P., Suhorutšenko J., Padari K. (2013). PepFect14 Peptide Vector for Efficient Gene Delivery in Cell Cultures. Mol. Pharm..

[131] Pärnaste L., Arukuusk P., Langel K., Tenson T., Langel Ü. (2017). The Formation of Nanoparticles between Small Interfering RNA and Amphipathic Cell-Penetrating Peptides. Mol. Ther. Nucleic Acids.

[132] Carreras-Badosa G., Maslovskaja J., Periyasamy K., Urgard E., Padari K., Vaher H., Tserel L., Gestin M., Kisand K., Arukuusk P. (2020). NickFect Type of Cell-Penetrating Peptides Present Enhanced Efficiency for MicroRNA-146a Delivery into Dendritic Cells and during Skin Inflammation. Biomaterials.

[133] Varkouhi A.K., Scholte M., Storm G., Haisma H.J. (2011). Endosomal Escape Pathways for Delivery of Biologicals. J. Control. Release.

[134] Mosquera J., García I., Liz-Marzán L.M. (2018). Cellular Uptake of Nanoparticles versus Small Molecules: A Matter of Size. Acc. Chem. Res..

[135] Behzadi S., Serpooshan V., Tao W., Hamaly M.A., Alkawareek M.Y., Dreaden E.C., Brown D., Alkilany A.M., Farokhzad O.C., Mahmoudi M. (2017). Cellular Uptake of Nanoparticles: Journey Inside the Cell. Chem. Soc. Rev..

[136] Lönn P., Kacsinta A.D., Cui X.-S., Hamil A.S., Kaulich M., Gogoi K., Dowdy S.F. (2016). Enhancing Endosomal Escape for Intracellular Delivery of Macromolecular Biologic Therapeutics. Sci. Rep..

[137] Ahmad A., Khan J.M., Haque S. (2019). Strategies in the Design of Endosomolytic Agents for Facilitating Endosomal Escape in Nanoparticles. Biochimie.

[138] Erazo-Oliveras A., Muthukrishnan N., Baker R., Wang T.-Y., Pellois J.-P. (2012). Improving the Endosomal Escape of Cell-Penetrating Peptides and Their Cargos: Strategies and Challenges. Pharmaceuticals.

[139] Daussy C.F., Wodrich H. (2020). “Repair Me If You Can”: Membrane Damage, Response, and Control from the Viral Perspective. Cells.

[140] Alhakamy N.A., Nigatu A.S., Berkland C.J., Ramsey J.D. (2013). Noncovalently Associated -Penetrating Peptides for Gene Delivery Applications. Ther. Deliv..

[141] Loughran S.P., McCrudden C.M., McCarthy H.O. (2015). Designer Peptide Delivery Systems for Gene Therapy. Eur. J. Nanomed..

[142] Sudo K., Niikura K., Iwaki K., Kohyama S., Fujiwara K., Doi N. (2017). Human-Derived Fusogenic Peptides for the Intracellular Delivery of Proteins. J. Control. Release.

[143] Ferrer-Miralles N., Vázquez E., Villaverde A. (2008). Membrane-Active Peptides for Non-Viral Gene Therapy: Making the Safest Easier. Trends Biotechnol..

[144] Wadia J.S., Stan R.V., Dowdy S.F. (2004). Transducible TAT-HA Fusogenic Peptide Enhances Escape of TAT-Fusion Proteins after Lipid Raft Macropinocytosis. Nat. Med..

[145] Ye S., Tian M., Wang T., Ren L., Wang D., Shen L., Shang T. (2012). Synergistic Effects of Cell-Penetrating Peptide Tat and Fusogenic Peptide HA2-Enhanced Cellular Internalization and Gene Transduction of Organosilica Nanoparticles. Nanomed. Nanotechnol. Biol. Med..

[146] Karjoo Z., McCarthy H.O., Patel P., Nouri F.S., Hatefi A. (2013). Systematic Engineering of Uniform, Highly Efficient, Targeted and Shielded Viral-Mimetic Nanoparticles. Small.

[147] Zhao X., Wang J., Tao S., Ye T., Kong X., Ren L. (2016). In Vivo Bio-Distribution and Efficient Tumor Targeting of Gelatin/Silica Nanoparticles for Gene Delivery. Nanoscale Res. Lett..

[148] Golan M., Feinshtein V., David A. (2016). Conjugates of HA2 with Octaarginine-Grafted HPMA Copolymer Offer Effective SiRNA Delivery and Gene Silencing in Cancer Cells. Eur. J. Pharm. Biopharm..

[149] Cantini L., Attaway C.C., Butler B., Andino L.M., Sokolosky M.L., Jakymiw A. (2013). Fusogenic-Oligoarginine Peptide-Mediated Delivery of SiRNAs Targeting the CIP2A Oncogene into Oral Cancer Cells. PLoS ONE.

[150] Funhoff A.M., van Nostrum C.F., Koning G.A., Schuurmans-Nieuwenbroek N.M.E., Crommelin D.J.A., Hennink W.E. (2004). Endosomal Escape of Polymeric Gene Delivery Complexes Is Not Always Enhanced by Polymers Buffering at Low PH. Biomacromolecules.

[151] Wang Y., Mangipudi S.S., Canine B.F., Hatefi A. (2009). A Designer Biomimetic Vector with a Chimeric Architecture for Targeted Gene Transfer. J. Control. Release Off. J. Control. Release Soc..

[152] Hou K.K., Pan H., Schlesinger P.H., Wickline S.A. (2015). A Role for Peptides in Overcoming Endosomal Entrapment in SiRNA Delivery—A Focus on Melittin. Biotechnol. Adv..

[153] Raghuraman H., Chattopadhyay A. (2007). Melittin: A Membrane-Active Peptide with Diverse Functions. Biosci. Rep..

[154] Paray B.A., Ahmad A., Khan J.M., Taufiq F., Pathan A., Malik A., Ahmed M.Z. (2021). The Role of the Multifunctional Antimicrobial Peptide Melittin in Gene Delivery. Drug Discov. Today.

[155] Schellinger J.G., Pahang J.A., Johnson R.N., Chu D.S.H., Sellers D.L., Maris D.O., Convertine A.J., Stayton P.S., Horner P.J., Pun S.H. (2013). Melittin-Grafted HPMA-Oligolysine Based Copolymers for Gene Delivery. Biomaterials.

[156] Sun Y., Yang Z., Wang C., Yang T., Cai C., Zhao X., Yang L., Ding P. (2017). Exploring the Role of Peptides in Polymer-Based Gene Delivery. Acta Biomater..

[157] Nouri F.S., Wang X., Dorrani M., Karjoo Z., Hatefi A. (2013). A Recombinant Biopolymeric Platform for Reliable Evaluation of the Activity of PH-Responsive Amphiphile Fusogenic Peptides. Biomacromolecules.

[158] Lee H., Jeong J.H., Park T.G. (2002). PEG Grafted Polylysine with Fusogenic Peptide for Gene Delivery: High Transfection Efficiency with Low Cytotoxicity. J. Control. Release.

[159] Bennett R., Yakkundi A., McKeen H.D., McClements L., McKeogh T.J., McCrudden C.M., Arthur K., Robson T., McCarthy H.O. (2015). RALA-Mediated Delivery of FKBPL Nucleic Acid Therapeutics. Nanomedicine.

[160] Mulholland E.J., Ali A., Robson T., Dunne N.J., McCarthy H.O. (2019). Delivery of RALA/SiFKBPL Nanoparticles via Electrospun Bilayer Nanofibres: An Innovative Angiogenic Therapy for Wound Repair. J. Control. Release.

[161] Yan L.-P., Castaño I.M., Sridharan R., Kelly D., Lemoine M., Cavanagh B.L., Dunne N.J., McCarthy H.O., O’Brien F.J. (2020). Collagen/GAG Scaffolds Activated by RALA-SiMMP-9 Complexes with Potential for Improved Diabetic Foot Ulcer Healing. Mater. Sci. Eng. C.

[162] Akita H., Masuda T., Nishio T., Niikura K., Ijiro K., Harashima H. (2011). Improving in Vivo Hepatic Transfection Activity by Controlling Intracellular Trafficking: The Function of GALA and Maltotriose. Mol. Pharm..

[163] Li X., Chen Y., Wang M., Ma Y., Xia W., Gu H. (2013). A Mesoporous Silica Nanoparticle–PEI–Fusogenic Peptide System for SiRNA Delivery in Cancer Therapy. Biomaterials.

[164] Ali A.A., McCrudden C.M., McCaffrey J., McBride J.W., Cole G., Dunne N.J., Robson T., Kissenpfennig A., Donnelly R.F., McCarthy H.O. (2017). DNA Vaccination for Cervical Cancer; a Novel Technology Platform of RALA Mediated Gene Delivery via Polymeric Microneedles. Nanomed. Nanotechnol. Biol. Med..

[165] McCrudden C.M., McBride J.W., McCaffrey J., McErlean E.M., Dunne N.J., Kett V.L., Coulter J.A., Robson T., McCarthy H.O. (2018). Gene Therapy with RALA/INOS Composite Nanoparticles Significantly Enhances Survival in a Model of Metastatic Prostate Cancer. Cancer Nanotechnol..

[166] Sousa Â., Almeida A.M., Faria R., Konate K., Boisguerin P., Queiroz J.A., Costa D. (2019). Optimization of Peptide-Plasmid DNA Vectors Formulation for Gene Delivery in Cancer Therapy Exploring Design of Experiments. Colloids Surf. B Biointerfaces.

[167] Gottschalk S., Sparrow J.T., Hauer J., Mims M.P., Leland F.E., Woo S.L.-Y., Smith L.C. (1996). A Novel DNA-Peptide Complex for Efficient Gene Transfer and Expression in Mammalian Cells. Gene Ther..

[168] Guryanov I.A., Vlasov G.P., Lesina E.A., Kiselev A.V., Baranov V.S., Avdeeva E.V., Vorob’ev V.I. (2005). Cationic Oligopeptides Modified with Lipophilic Fragments: Use for DNA Delivery to Cells. Russ. J. Bioorg. Chem..

[169] Van Rossenberg S.M.W., Sliedregt-Bol K.M., Meeuwenoord N.J., van Berkel T.J.C., van Boom J.H., van der Marel G.A., Biessen E.A.L. (2002). Targeted Lysosome Disruptive Elements for Improvement of Parenchymal Liver Cell-Specific Gene Delivery. J. Biol. Chem..

[170] Meng Z., Luan L., Kang Z., Feng S., Meng Q., Liu K. (2017). Histidine-Enriched Multifunctional Peptide Vectors with Enhanced Cellular Uptake and Endosomal Escape for Gene Delivery. J. Mater. Chem. B.

[171] Kichler A., Mason A.J., Bechinger B. (2006). Cationic Amphipathic Histidine-Rich Peptides for Gene Delivery. Biochim. Biophys. Acta BBA Biomembr..

[172] Midoux P., Pichon C., Yaouanc J.-J., Jaffrès P.-A. (2009). Chemical Vectors for Gene Delivery: A Current Review on Polymers, Peptides and Lipids Containing Histidine or Imidazole as Nucleic Acids Carriers. Br. J. Pharmacol..

[173] Chen Q.-R., Zhang L., Stass S.A., Mixson A.J. (2000). Co-Polymer of Histidine and Lysine Markedly Enhances Transfection Efficiency of Liposomes. Gene Ther..

[174] Pichon C., Gonçalves C., Midoux P. (2001). Histidine-Rich Peptides and Polymers for Nucleic Acids Delivery. Adv. Drug Deliv. Rev..

[175] Perche F., Gosset D., Mével M., Miramon M.-L., Yaouanc J.-J., Pichon C., Benvegnu T., Jaffrès P.-A., Midoux P. (2011). Selective Gene Delivery in Dendritic Cells with Mannosylated and Histidylated Lipopolyplexes. J. Drug Target..

[176] Perche F., Benvegnu T., Berchel M., Lebegue L., Pichon C., Jaffrès P.-A., Midoux P. (2011). Enhancement of Dendritic Cells Transfection in Vivo and of Vaccination against B16F10 Melanoma with Mannosylated Histidylated Lipopolyplexes Loaded with Tumor Antigen Messenger RNA. Nanomed. Nanotechnol. Biol. Med..

[177] Perche F., Lambert O., Berchel M., Jaffrès P.-A., Pichon C., Midoux P. (2012). Gene Transfer by Histidylated Lipopolyplexes: A Dehydration Method Allowing Preservation of Their Physicochemical Parameters and Transfection Efficiency. Int. J. Pharm..

[178] Zhu H., Dong C., Dong H., Ren T., Wen X., Su J., Li Y. (2014). Cleavable PEGylation and Hydrophobic Histidylation of Polylysine for SiRNA Delivery and Tumor Gene Therapy. ACS Appl. Mater. Interfaces.

[179] Zhu J., Qiao M., Wang Q., Ye Y., Ba S., Ma J., Hu H., Zhao X., Chen D. (2018). Dual-Responsive Polyplexes with Enhanced Disassembly and Endosomal Escape for Efficient Delivery of SiRNA. Biomaterials.

[180] Sadeghian F., Hosseinkhani S., Alizadeh A., Hatefi A. (2012). Design, Engineering and Preparation of a Multi-Domain Fusion Vector for Gene Delivery. Int. J. Pharm..

[181] Asseline U., Gonçalves C., Pichon C., Midoux P. (2014). Improved Nuclear Delivery of Antisense 2’-Ome RNA by Conjugation with the Histidine-Rich Peptide H5WYG. J. Gene Med..

[182] McErlean E.M., McCrudden C.M., McCarthy H.O. (2016). Delivery of Nucleic Acids for Cancer Gene Therapy: Overcoming Extra- and Intra-Cellular Barriers. Ther. Deliv..

[183] McBride J.W., Massey A.S., McCaffrey J., McCrudden C.M., Coulter J.A., Dunne N.J., Robson T., McCarthy H.O. (2016). Development of TMTP-1 Targeted Designer Biopolymers for Gene Delivery to Prostate Cancer. Int. J. Pharm..

[184] Alipour M., Hosseinkhani S., Sheikhnejad R., Cheraghi R. (2017). Nano-Biomimetic Carriers Are Implicated in Mechanistic Evaluation of Intracellular Gene Delivery. Sci. Rep..

[185] Alipour M., Majidi A., Molaabasi F., Sheikhnejad R., Hosseinkhani S. (2018). In Vivo Tumor Gene Delivery Using Novel Peptideticles: PH-Responsive and Ligand Targeted Core–Shell Nanoassembly. Int. J. Cancer.

[186] Thapa R.K., Sullivan M.O. (2018). Gene Delivery by Peptide-Assisted Transport. Curr. Opin. Biomed. Eng..

[187] Liu J., Guo N., Gao C., Liu N., Zheng X., Tan Y., Lei J., Hao Y., Chen L., Zhang X. (2019). Effective Gene Silencing Mediated by Polypeptide Nanoparticles LAH4-L1-SiMDR1 in Multi-Drug Resistant Human Breast Cancer. J. Biomed. Nanotechnol..

[188] Liang W., Lam J.K.W. (2012). Endosomal Escape Pathways for Non-Viral Nucleic Acid Delivery Systems.

[189] Bechinger B. (1996). Towards Membrane Protein Design: PH-Sensitive Topology of Histidine-Containing Polypeptides. J. Mol. Biol..

[190] Perrone B., Miles A.J., Salnikov E.S., Wallace B.A., Bechinger B. (2014). Lipid Interactions of LAH4, a Peptide with Antimicrobial and Nucleic Acid Transfection Activities. Eur. Biophys. J..

[191] Marquette A., Mason A.J., Bechinger B. (2008). Aggregation and Membrane Permeabilizing Properties of Designed Histidine-Containing Cationic Linear Peptide Antibiotics. J. Pept. Sci. Off. Publ. Eur. Pept. Soc..

[192] Wolf J., Aisenbrey C., Harmouche N., Raya J., Bertani P., Voievoda N., Süss R., Bechinger B. (2017). PH-Dependent Membrane Interactions of the Histidine-Rich Cell-Penetrating Peptide LAH4-L1. Biophys. J..

[193] Langlet-Bertin B., Leborgne C., Scherman D., Bechinger B., Mason A.J., Kichler A. (2010). Design and Evaluation of Histidine-Rich Amphipathic Peptides for SiRNA Delivery. Pharm. Res..

[194] Xu Y., Liang W., Qiu Y., Cespi M., Palmieri G.F., Mason A.J., Lam J.K.W. (2016). Incorporation of a Nuclear Localization Signal in PH Responsive LAH4-L1 Peptide Enhances Transfection and Nuclear Uptake of Plasmid DNA. Mol. Pharm..

[195] Moulay G., Leborgne C., Mason A.J., Aisenbrey C., Kichler A., Bechinger B. (2017). Histidine-Rich Designer Peptides of the LAH4 Family Promote Cell Delivery of a Multitude of Cargo. J. Pept. Sci..

[196] Liu N., Bechinger B., Süss R. (2017). The Histidine-Rich Peptide LAH4-L1 Strongly Promotes PAMAM-Mediated Transfection at Low Nitrogen to Phosphorus Ratios in the Presence of Serum. Sci. Rep..

[197] Kichler A., Mason A.J., Marquette A., Bechinger B., Ogris M., Sami H. (2019). Histidine-Rich Cationic Cell-Penetrating Peptides for Plasmid DNA and siRNA Delivery. Nanotechnology for Nucleic Acid Delivery: Methods and Protocols.

[198] Marquette A., Leborgne C., Schartner V., Salnikov E., Bechinger B., Kichler A. (2020). Peptides Derived from the C-Terminal Domain of HIV-1 Viral Protein R in Lipid Bilayers: Structure, Membrane Positioning and Gene Delivery. Biochim. Biophys. Acta BBA Biomembr..

[199] Chamarthy S.P., Kovacs J.R., McClelland E., Gattens D., Meng W.S. (2003). A Cationic Peptide Consists of Ornithine and Histidine Repeats Augments Gene Transfer in Dendritic Cells. Mol. Immunol..

[200] Kovacs J.R., Zheng Y., Shen H., Meng W.S. (2005). Polymeric Microspheres as Stabilizing Anchors for Oligonucleotide Delivery to Dendritic Cells. Biomaterials.

[201] Jia L., Kovacs J.R., Zheng Y., Gawalt E.S., Shen H., Meng W.S. (2006). Attenuated Alloreactivity of Dendritic Cells Engineered with Surface-Modified Microspheres Carrying a Plasmid Encoding Interleukin-10. Biomaterials.

[202] Lo S.L., Wang S. (2008). An Endosomolytic Tat Peptide Produced by Incorporation of Histidine and Cysteine Residues as a Nonviral Vector for DNA Transfection. Biomaterials.

[203] Hao X., Li Q., Ali H., Zaidi S.S.A., Guo J., Ren X., Shi C., Xia S., Zhang W., Feng Y. (2018). POSS-Cored and Peptide Functionalized Ternary Gene Delivery Systems with Enhanced Endosomal Escape Ability for Efficient Intracellular Delivery of Plasmid DNA. J. Mater. Chem. B.

[204] Li Q., Hao X., Zaidi S.S.A., Guo J., Ren X., Shi C., Zhang W., Feng Y. (2018). Oligohistidine and Targeting Peptide Functionalized TAT-NLS for Enhancing Cellular Uptake and Promoting Angiogenesis in Vivo. J. Nanobiotechnol..

[205] Read M.L., Singh S., Ahmed Z., Stevenson M., Briggs S.S., Oupicky D., Barrett L.B., Spice R., Kendall M., Berry M. (2005). A Versatile Reducible Polycation-Based System for Efficient Delivery of a Broad Range of Nucleic Acids. Nucleic Acids Res..

[206] Stevenson M., Ramos-Perez V., Singh S., Soliman M., Preece J.A., Briggs S.S., Read M.L., Seymour L.W. (2008). Delivery of SiRNA Mediated by Histidine-Containing Reducible Polycations. J. Control. Release Off. J. Control. Release Soc..

[207] Durymanov M., Reineke J. (2018). Non-Viral Delivery of Nucleic Acids: Insight Into Mechanisms of Overcoming Intracellular Barriers. Front. Pharmacol..

[208] Lin G., Li L., Panwar N., Wang J., Tjin S.C., Wang X., Yong K.-T. (2018). Non-Viral Gene Therapy Using Multifunctional Nanoparticles: Status, Challenges, and Opportunities. Coord. Chem. Rev..

[209] Uludag H., Ubeda A., Ansari A. (2019). At the Intersection of Biomaterials and Gene Therapy: Progress in Non-Viral Delivery of Nucleic Acids. Front. Bioeng. Biotechnol..

[210] Vermeulen L.M.P., Brans T., De Smedt S.C., Remaut K., Braeckmans K. (2018). Methodologies to Investigate Intracellular Barriers for Nucleic Acid Delivery in Non-Viral Gene Therapy. Nano Today.

[211] Shi B., Zheng M., Tao W., Chung R., Jin D., Ghaffari D., Farokhzad O.C. (2017). Challenges in DNA Delivery and Recent Advances in Multifunctional Polymeric DNA Delivery Systems. Biomacromolecules.

[212] Yao J., Fan Y., Li Y., Huang L. (2013). Strategies on the Nuclear-Targeted Delivery of Genes. J. Drug Target..

[213] Zelmer C., Zweifel L.P., Kapinos L.E., Craciun I., Güven Z.P., Palivan C.G., Lim R.Y.H. (2020). Organelle-Specific Targeting of Polymersomes into the Cell Nucleus. Proc. Natl. Acad. Sci. USA.

[214] Kosugi S., Hasebe M., Matsumura N., Takashima H., Miyamoto-Sato E., Tomita M., Yanagawa H. (2009). Six Classes of Nuclear Localization Signals Specific to Different Binding Grooves of Importin α. J. Biol. Chem..

[215] Pan L., He Q., Liu J., Chen Y., Ma M., Zhang L., Shi J. (2012). Nuclear-Targeted Drug Delivery of TAT Peptide-Conjugated Monodisperse Mesoporous Silica Nanoparticles. J. Am. Chem. Soc..

[216] Van der Aa M.A.E.M., Mastrobattista E., Oosting R.S., Hennink W.E., Koning G.A., Crommelin D.J.A. (2006). The Nuclear Pore Complex: The Gateway to Successful Nonviral Gene Delivery. Pharm. Res..

[217] Cartier R., Reszka R. (2002). Utilization of Synthetic Peptides Containing Nuclear Localization Signals for Nonviral Gene Transfer Systems. Gene Ther..

[218] Escriou V., Carrière M., Scherman D., Wils P. (2003). NLS Bioconjugates for Targeting Therapeutic Genes to the Nucleus. Adv. Drug Deliv. Rev..

[219] Zhao M., Li J., Ji H., Chen D., Hu H. (2019). A Versatile Endosome Acidity-Induced Sheddable Gene Delivery System: Increased Tumor Targeting and Enhanced Transfection Efficiency. Int. J. Nanomedicine.

[220] Dean D., Strong D., Zimmer W. (2005). Nuclear Entry of Nonviral Vectors. Gene Ther..

[221] Morille M., Passirani C., Vonarbourg A., Clavreul A., Benoit J.-P. (2008). Progress in Developing Cationic Vectors for Non-Viral Systemic Gene Therapy against Cancer. Biomaterials.

[222] Bogacheva M., Egorova A., Slita A., Maretina M., Baranov V., Kiselev A. (2017). Arginine-Rich Cross-Linking Peptides with Different SV40 Nuclear Localization Signal Content as Vectors for Intranuclear DNA Delivery. Bioorg. Med. Chem. Lett..

[223] Siomi H., Dreyfuss G. (1995). A Nuclear Localization Domain in the HnRNP A1 Protein. J. Cell Biol..

[224] Bremner K.H., Seymour L.W., Logan A., Read M.L. (2004). Factors Influencing the Ability of Nuclear Localization Sequence Peptides To Enhance Nonviral Gene Delivery. Bioconjug. Chem..

[225] Nicolson G.L. (2014). Mitochondrial Dysfunction and Chronic Disease: Treatment With Natural Supplements. Integr. Med. Encinitas Calif.

[226] Tiku V., Tan M.-W., Dikic I. (2020). Mitochondrial Functions in Infection and Immunity. Trends Cell Biol..

[227] Gallay P., Stitt V., Mundy C., Oettinger M., Trono D. (1996). Role of the Karyopherin Pathway in Human Immunodeficiency Virus Type 1 Nuclear Import. J. Virol..

[228] Robbins J., Dilwortht S.M., Laskey R.A., Dingwall C. (1991). Two Interdependent Basic Domains in Nucleoplasmin Nuclear Targeting Sequence: Identification of a Class of Bipartite Nuclear Targeting Sequence. Cell.

[229] Donkuru M., Badea I., Wettig S., Verrall R., Elsabahy M., Foldvari M. (2010). Advancing Nonviral Gene Delivery: Lipid- and Surfactant-Based Nanoparticle Design Strategies. Nanomedicine.

[230] Preuss M., Tecle M., Shah I., Matthews D.A., D. Miller A. (2003). Comparison between the Interactions of Adenovirus-Derived Peptides with Plasmid DNA and Their Role in Gene Delivery Mediated by Liposome–Peptide–DNA Virus-like Nanoparticles. Org. Biomol. Chem..

[231] Hébert E. (2003). Improvement of Exogenous DNA Nuclear Importation by Nuclear Localization Signal-Bearing Vectors: A Promising Way for Non-Viral Gene Therapy?. Biol. Cell.

[232] Li Q., Huang Y. (2020). Mitochondrial Targeted Strategies and Theirapplication for Cancer and Other Diseases Treatment. J. Pharm. Investig..

[233] Kim S., Nam H.Y., Lee J., Seo J. (2020). Mitochondrion-Targeting Peptides and Peptidomimetics: Recent Progress and Design Principles. Biochemistry.

[234] Jang Y., Lim K. (2018). Recent Advances in Mitochondria-Targeted Gene Delivery. Mol. J. Synth. Chem. Nat. Prod. Chem..

[235] Wang H., Fang B., Peng B., Wang L., Xue Y., Bai H., Lu S., Voelcker N.H., Li L., Fu L. (2021). Recent Advances in Chemical Biology of Mitochondria Targeting. Front. Chem..

[236] Kang Y.C., Son M., Kang S., Im S., Piao Y., Lim K.S., Song M.-Y., Park K.-S., Kim Y.-H., Pak Y.K. (2018). Cell-Penetrating Artificial Mitochondria-Targeting Peptide-Conjugated Metallothionein 1A Alleviates Mitochondrial Damage in Parkinson’s Disease Models. Exp. Mol. Med..

[237] Horton K.L., Stewart K.M., Fonseca S.B., Guo Q., Kelley S.O. (2008). Mitochondria-Penetrating Peptides. Chem. Biol..

[238] Horne W.S., Stout C.D., Ghadiri M.R. (2003). A Heterocyclic Peptide Nanotube. J. Am. Chem. Soc..

[239] Wu J., Li J., Wang H., Liu C.-B. (2018). Mitochondrial-Targeted Penetrating Peptide Delivery for Cancer Therapy. Expert Opin. Drug Deliv..

[240] Lin R., Zhang P., Cheetham A.G., Walston J., Abadir P., Cui H. (2015). Dual Peptide Conjugation Strategy for Improved Cellular Uptake and Mitochondria Targeting. Bioconjug. Chem..

[241] Ezzat K., EL Andaloussi S., Zaghloul E.M., Lehto T., Lindberg S., Moreno P.M.D., Viola J.R., Magdy T., Abdo R., Guterstam P. (2011). PepFect 14, a Novel Cell-Penetrating Peptide for Oligonucleotide Delivery in Solution and as Solid Formulation. Nucleic Acids Res..

[242] Cerrato C.P., Kivijärvi T., Tozzi R., Lehto T., Gestin M., Langel Ü. (2020). Intracellular Delivery of Therapeutic Antisense Oligonucleotides Targeting MRNA Coding Mitochondrial Proteins by Cell-Penetrating Peptides. J. Mater. Chem. B.

[243] Deshayes S., Konate K., Dussot M., Chavey B., Vaissière A., Van T.N.N., Aldrian G., Padari K., Pooga M., Vivès E. (2020). Deciphering the Internalization Mechanism of WRAP:SiRNA Nanoparticles. Biochim. Biophys. Acta BBA Biomembr..

[244] Faria R., Vivés E., Boisguerin P., Sousa A., Costa D. (2021). Development of Peptide-Based Nanoparticles for Mitochondrial Plasmid DNA Delivery. Polymers.

[245] Jain A., Chugh A. (2016). Mitochondrial Transit Peptide Exhibits Cell Penetration Ability and Efficiently Delivers Macromolecules to Mitochondria. FEBS Lett..

[246] Fatouros D.G., Lamprou D.A., Urquhart A.J., Yannopoulos S.N., Vizirianakis I.S., Zhang S., Koutsopoulos S. (2014). Lipid-like Self-Assembling Peptide Nanovesicles for Drug Delivery. ACS Appl. Mater. Interfaces.

[247] Liang J., Wu W.-L., Xu X.-D., Zhuo R.-X., Zhang X.-Z. (2014). PH Responsive Micelle Self-Assembled from a New Amphiphilic Peptide as Anti-Tumor Drug Carrier. Colloids Surf. B Biointerfaces.

[248] Wang Q., Zhang X., Zheng J., Liu D. (2014). Self-Assembled Peptide Nanotubes as Potential Nanocarriers for Drug Delivery. RSC Adv..

[249] Zhang C., Xue X., Luo Q., Li Y., Yang K., Zhuang X., Jiang Y., Zhang J., Liu J., Zou G. (2014). Self-Assembled Peptide Nanofibers Designed as Biological Enzymes for Catalyzing Ester Hydrolysis. ACS Nano.

[250] Wang M., Wang J., Zhou P., Deng J., Zhao Y., Sun Y., Yang W., Wang D., Li Z., Hu X. (2018). Nanoribbons Self-Assembled from Short Peptides Demonstrate the Formation of Polar Zippers between β-Sheets. Nat. Commun..

[251] Pereira-Silva M., Jarak I., Alvarez-Lorenzo C., Concheiro A., Santos A.C., Veiga F., Figueiras A. (2020). Micelleplexes as Nucleic Acid Delivery Systems for Cancer-Targeted Therapies. J. Control. Release.

[252] Ibaraki H., Kanazawa T., Owada M., Iwaya K., Takashima Y., Seta Y. (2020). Anti-Metastatic Effects on Melanoma via Intravenous Administration of Anti-NF-ΚB SiRNA Complexed with Functional Peptide-Modified Nano-Micelles. Pharmaceutics.

[253] Zhang Y., Wang Y., Meng L., Huang Q., Zhu Y., Cui W., Cheng Y., Liu R. (2021). Targeted Micelles with Chemotherapeutics and Gene Drugs to Inhibit the G1/S and G2/M Mitotic Cycle of Prostate Cancer. J. Nanobiotechnol..

[254] Mazza M., Hadjidemetriou M., de Lázaro I., Bussy C., Kostarelos K. (2015). Peptide Nanofiber Complexes with SiRNA for Deep Brain Gene Silencing by Stereotactic Neurosurgery. ACS Nano.

[255] Avila L.A., Aps L.R.M.M., Sukthankar P., Ploscariu N., Gudlur S., Šimo L., Szoszkiewicz R., Park Y., Lee S.Y., Iwamoto T. (2015). Branched Amphiphilic Cationic Oligopeptides Form Peptiplexes with DNA: A Study of Their Biophysical Properties and Transfection Efficiency. Mol. Pharm..

[256] Abedi-Gaballu F., Dehghan G., Ghaffari M., Yekta R., Abbaspour-Ravasjani S., Baradaran B., Dolatabadi J.E.N., Hamblin M.R. (2018). PAMAM Dendrimers as Efficient Drug and Gene Delivery Nanosystems for Cancer Therapy. Appl. Mater. Today.

[257] Gorzkiewicz M., Konopka M., Janaszewska A., Tarasenko I.I., Sheveleva N.N., Gajek A., Neelov I.M., Klajnert-Maculewicz B. (2020). Application of New Lysine-Based Peptide Dendrimers D3K2 and D3G2 for Gene Delivery: Specific Cytotoxicity to Cancer Cells and Transfection in Vitro. Bioorg. Chem..

[258] Bahadoran A., Moeini H., Bejo M.H., Hussein M.Z., Omar A.R. (2016). Development of Tat-Conjugated Dendrimer for Transdermal DNA Vaccine Delivery. J. Pharm. Pharm. Sci..

[259] Yang L., Tang H., Sun H. (2018). Progress in Photo-Responsive Polypeptide Derived Nano-Assemblies. Micromachines.

[260] Mura S., Nicolas J., Couvreur P. (2013). Stimuli-Responsive Nanocarriers for Drug Delivery. Nat. Mater..

[261] Lee D., Rejinold N.S., Jeong S.D., Kim Y.-C. (2018). Stimuli-Responsive Polypeptides for Biomedical Applications. Polymers.

[262] Han K., Chen S., Chen W.-H., Lei Q., Liu Y., Zhuo R.-X., Zhang X.-Z. (2013). Synergistic Gene and Drug Tumor Therapy Using a Chimeric Peptide. Biomaterials.

[263] Levine M.N., Raines R.T. (2012). Trimethyl Lock: A Trigger for Molecular Release in Chemistry, Biology, and Pharmacology. Chem. Sci..

[264] Kuppusamy P., Li H., Ilangovan G., Cardounel A.J., Zweier J.L., Yamada K., Krishna M.C., Mitchell J.B. (2002). Noninvasive Imaging of Tumor Redox Status and Its Modification by Tissue Glutathione Levels. Cancer Res..

[265] Cao C.-Y., Shen Y.-Y., Wang J.-D., Li L., Liang G.-L. (2013). Controlled Intracellular Self-Assembly of Gadolinium Nanoparticles as Smart Molecular MR Contrast Agents. Sci. Rep..

[266] Löwik D.W.P.M., Leunissen E.H.P., van den Heuvel M., Hansen M.B., van Hest J.C.M. (2010). Stimulus Responsive Peptide Based Materials. Chem. Soc. Rev..

[267] Mackay J.A., Chilkoti A. (2008). Temperature Sensitive Peptides: Engineering Hyperthermia-Directed Therapeutics. Int. J. Hyperthermia.

[268] Sigg S.J., Postupalenko V., Duskey J.T., Palivan C.G., Meier W. (2016). Stimuli-Responsive Codelivery of Oligonucleotides and Drugs by Self-Assembled Peptide Nanoparticles. Biomacromolecules.

[269] Ouboter D.d.B., Schuster T., Shanker V., Heim M., Meier W. (2014). Multicompartment Micelle-Structured Peptide Nanoparticles: A New Biocompatible Gene- and Drug-Delivery Tool. J. Biomed. Mater. Res. A.

[270] Abbas M., Zou Q., Li S., Yan X. (2017). Self-Assembled Peptide- and Protein-Based Nanomaterials for Antitumor Photodynamic and Photothermal Therapy. Adv. Mater..

[271] Ding J., Zhuang X., Xiao C., Cheng Y., Zhao L., He C., Tang Z., Chen X. (2011). Preparation of Photo-Cross-Linked PH-Responsive Polypeptide Nanogels as Potential Carriers for Controlled Drug Delivery. J. Mater. Chem..

[272] Liu G., Dong C.-M. (2012). Photoresponsive Poly( S -( o -Nitrobenzyl)- l -Cysteine)- b -PEO from a l -Cysteine N -Carboxyanhydride Monomer: Synthesis, Self-Assembly, and Phototriggered Drug Release. Biomacromolecules.

[273] Yang Y., Xia X., Dong W., Wang H., Li L., Ma P., Sheng W., Xu X., Liu Y. (2016). Acid Sensitive Polymeric Micelles Combining Folate and Bioreducible Conjugate for Specific Intracellular SiRNA Delivery. Macromol. Biosci..

[274] Hu Q., Wang K., Sun X., Li Y., Fu Q., Liang T., Tang G. (2016). A Redox-Sensitive, Oligopeptide-Guided, Self-Assembling, and Efficiency-Enhanced (ROSE) System for Functional Delivery of MicroRNA Therapeutics for Treatment of Hepatocellular Carcinoma. Biomaterials.

[275] Wang H.-X., Song Z., Lao Y.-H., Xu X., Gong J., Cheng D., Chakraborty S., Park J.S., Li M., Huang D. (2018). Nonviral Gene Editing via CRISPR/Cas9 Delivery by Membrane-Disruptive and Endosomolytic Helical Polypeptide. Proc. Natl. Acad. Sci. USA.

[276] Reissmann S., Filatova M.P. (2021). New Generation of Cell-Penetrating Peptides: Functionality and Potential Clinical Application. J. Pept. Sci..

[277] Hallan S.S., Sguizzato M., Esposito E., Cortesi R. (2021). Challenges in the Physical Characterization of Lipid Nanoparticles. Pharmaceutics.

[278] Aldosari B.N., Alfagih I.M., Almurshedi A.S. (2021). Lipid Nanoparticles as Delivery Systems for RNA-Based Vaccines. Pharmaceutics.

[279] Reese H.R., Shanahan C.C., Proulx C., Menegatti S. (2020). Peptide Science: A “Rule Model” for New Generations of Peptidomimetics. Acta Biomater..

[280] Qvit N., Rubin S.J.S., Urban T.J., Mochly-Rosen D., Gross E.R. (2017). Peptidomimetic Therapeutics: Scientific Approaches and Opportunities. Drug Discov. Today.

[281] Zhang G., Sun H.J. (2014). Racemization in Reverse: Evidence That D-Amino Acid Toxicity on Earth Is Controlled by Bacteria with Racemases. PLoS ONE.

